# Low expression of miR-182 caused by DNA hypermethylation accelerates acute lymphocyte leukemia development by targeting PBX3 and BCL2: miR-182 promoter methylation is a predictive marker for hypomethylation agents + BCL2 inhibitor venetoclax

**DOI:** 10.1186/s13148-024-01658-2

**Published:** 2024-03-26

**Authors:** Danyang Li, Yigang Yuan, Chen Meng, Zihan Lin, Min Zhao, Liuzhi Shi, Min Li, Daijiao Ye, Yue Cai, Xiaofei He, Haige Ye, Shujuan Zhou, Haixia Zhou, Shenmeng Gao

**Affiliations:** 1 Medical Research Center, The First Affiliated Hospital of Wenzhou Medical University, 1 Xuefubei Street, Ouhai District, Wenzhou, 325000 Zhejiang Province China; 2 Department of Clinical Laboratory, The First Affiliated Hospital of Wenzhou Medical University, 1 Xuefubei Street, Ouhai District, Wenzhou, Zhejiang Province China; 3 Department of Clinical Medicine, Wenzhou Medical University, Chashan District, Wenzhou, Zhejiang Province China; 4 The Key Laboratory of Pediatric Hematology and Oncology Diseases of Wenzhou, The Second Affiliated Hospital and Yuying Children’s Hospital of Wenzhou Medical University, 109 Xuanyuanxi Road, Wenzhou, Zhejiang Province China; 5 Department of Hematology, The First Affiliated Hospital of Wenzhou Medical University, 1 Xuefubei Street, Ouhai District, Wenzhou, Zhejiang Province China; 6 Department of Hematology, The Second Affiliated Hospital and Yuying Children’s Hospital of Wenzhou Medical University, 109 Xuanyuanxi Road, Wenzhou, Zhejiang Province China

**Keywords:** DNA hypermethylation, Hypomethylation agents, Acute lymphoblastic leukemia, microRNA, BCL2

## Abstract

**Background:**

miR-182 promoter hypermethylation frequently occurs in various tumors, including acute myeloid leukemia, and leads to low expression of miR-182. However, whether adult acute lymphocyte leukemia (ALL) cells have high miR-182 promoter methylation has not been determined.

**Methods:**

To assess the methylation status of the miR-182 promoter, methylation and unmethylation-specific PCR analysis, bisulfite-sequencing analysis, and MethylTarget™ assays were performed to measure the frequency of methylation at the miR-182 promoter. Bone marrow cells were isolated from miR-182 knockout (182KO) and 182 wild type (182WT) mice to construct BCR-ABL (P190) and Notch-induced murine B-ALL and T-ALL models, respectively. Primary ALL samples were performed to investigate synergistic effects of the hypomethylation agents (HMAs) and the BCL2 inhibitor venetoclax (Ven) in vitro.

**Results:**

miR-182 (miR-182-5P) expression was substantially lower in ALL blasts than in normal controls (NCs) because of DNA hypermethylation at the miR-182 promoter in ALL blasts but not in normal controls (NCs). Knockout of miR-182 (182KO) markedly accelerated ALL development, facilitated the infiltration, and shortened the OS in a BCR-ABL (P190)-induced murine B-ALL model. Furthermore, the 182KO ALL cell population was enriched with more leukemia-initiating cells (CD43^+^B220^+^ cells, LICs) and presented higher leukemogenic activity than the 182WT ALL population. Furthermore, depletion of miR-182 reduced the OS in a Notch-induced murine T-ALL model, suggesting that miR-182 knockout accelerates ALL development. Mechanistically, overexpression of miR-182 inhibited proliferation and induced apoptosis by directly targeting *PBX3* and *BCL2*, two well-known oncogenes, that are key targets of miR-182. Most importantly, DAC in combination with Ven had synergistic effects on ALL cells with miR-182 promoter hypermethylation, but not on ALL cells with miR-182 promoter hypomethylation.

**Conclusions:**

Collectively, we identified miR-182 as a tumor suppressor gene in ALL cells and low expression of miR-182 because of hypermethylation facilitates the malignant phenotype of ALL cells. DAC + Ven cotreatment might has been applied in the clinical try for ALL patients with miR-182 promoter hypermethylation. Furthermore, the methylation frequency at the miR-182 promoter should be a potential biomarker for DAC + Ven treatment in ALL patients.

**Supplementary Information:**

The online version contains supplementary material available at 10.1186/s13148-024-01658-2.

## Background

Acute lymphoblastic leukemia (B-ALL or T-ALL) is a severe malignant hematopoietic disorder originating from the uncontrollable clonal expansion of precursor B or T cells. Although ALL commonly occurs in children or youth and has high curability in most cases, high-risk and relapsed ALL patients, especially adult ALL patients, remain a therapeutic challenge [1]. Chemotherapy, bone marrow transplantation (BMT), chimeric antigen receptor T (CAR-T) cell therapy, or combinations of these treatments have markedly improved the OS of ALL patients [2, 3]. However, drug resistance, non-responsiveness, and severe side effects severely hinder the further clinical outcomes in ALL patients. Thus, novel therapeutic strategies targeting the oncogenic signaling pathway are urgently needed [4, 5].

MicroRNAs (miRNAs) are a class of small noncoding RNAs that post-transcriptionally regulate the expression of target genes by degrading or repressing the translation of target mRNAs. MiRNAs play essential regulatory roles in cancer cell survival, proliferation, invasion, and apoptosis [6]. Although several reports indicate that miR-182 can function as an oncogene [7, 8], multiple studies indicate that miR-182 functions as tumor suppressor gene [9–11]. For example, miR-182 suppresses tumorigenesis in colon cancer and clear cell renal cell carcinoma by inhibiting VEGF-C and UBE2T expression, respectively [10, 11]. Our report confirmed that miR-182 acts as a tumor suppressor gene by inducing apoptosis and inhibiting the self-renewal of leukemia stem cell (LSC) in acute myeloid cells. Furthermore, low expression of miR-182 caused by promoter hypermethylation facilitates the leukemogenesis [12]. However, the methylation status of miR-182 promoter and the exact function of miR-182 in ALL cells are largely unknown.

Aberrant epigenetic patterning is a hallmark of leukemic cells. DNA methylation at sequential cytosine-phosphate-guanine (CpG) dinucleotides disrupts promoter ability, resulting in the downregulation of tumor suppressor genes. Thus, hypermethylation of tumor suppressor genes frequently occurring in leukemic cells is associated with worse clinical outcomes in patients with myeloid and lymphoid malignancies[13, 14]. Hypomethylation agents (HMAs), such as decitabine (DAC) and 5'-Azacitidine (AZA), are extensively used to treat AML and myelodysplastic syndromes (MDS) through reducing global hypermethylation [15]. Although single HMA or Ven has a limited therapeutic response for ALL or AML patients, HMAs + Ven cotreatment substantially increases the response rate and improves the OS for elderly AML patients or untreated AML patients ineligible for intensive chemotherapy [16, 17] Thus, this combination treatment has been used as a first-line therapy for elderly AML patients [18]. However, whether HMAs + Ven cotreatment could be used in ALL patients and the exact predictors of response to HMAs + Ven remain elusive.

## Material and methods

### Cell lines, primary ALL blasts, and normal controls (NCs)

Human ALL cell lines, including NALM-6, REH, RS4;11, Molt-4, and Jurkat (ATCC, Manassas, VA, USA), were cultured in a humidified 37 °C incubator with 5% CO_2_ in RPMI 1640 medium supplemented with 10% fetal bovine serum (FBS; Sigma‒Aldrich, St. Louis, MO, USA) and 1% penicillin–streptomycin. Bone marrow (BM) mononuclear cells (MNCs) from untreated adult ALL patients (Additional file 1: Table S1) were obtained by density gradient centrifugation after obtaining the approval of the Ethics Committee of the First Affiliated Hospital of Wenzhou Medical University. Primary ALL blasts were cultured in StemSpan Serum-Free Expansion Medium (SFEM; Stemcell Technologies) supplemented with recombinant human interleukin-3 (IL-3, PeproTech Rocky Hill, NJ, USA), interleukin-7 (IL-6, PeproTech), and stem cell factor (SCF, PeproTech,) at 50 ng/mL each. Normal human BM MNCs were isolated from healthy volunteers by density gradient centrifugation as normal controls (NCs). All patients and healthy volunteers provided informed consent and all procedures in our studies were performed according to the Declaration of Helsinki and the Ethics Committee of the First Affiliated Hospital of Wenzhou Medical University.

### miRNA and mRNA extraction and quantitative real-time PCR (qRT-PCR)

Total mRNA and miRNA were extracted from human and murine ALL cells with Trizol reagent (Invitrogen, Carlsbad, CA, USA) according to the manufacturer's instruction with minor modifications[19]. The RNA concentration and quality were analyzed by measuring the absorbance at 260/280 nm with a spectrophotometer (DS-11, DeNovix, Wilmington, DE, USA). To measure the expression of miR-182 in ALL cells, U6 small nuclear RNA (snRNA) and miR-182 were reversely transcribed by Stem‒loop RT primers (RIBOBIO Company, Guangzhou, China) with PrimeScript™ RT Master Mix (Takara Bio, Tokyo, Japan). To measure mRNA expression, cDNA was synthesized by using total RNA as a template for qRT‒PCR analysis. SYBR Green dye (Vazyme Biotech, Nanjing, Jiangsu, China) was used to determine the expressions of U6, miR-182, and mRNA by ABI 7500 real-time PCR system (Applied Biosystems, Carlsbad, CA, USA). Relative expression was calculated using the 2^−ΔΔCT^ method. GAPDH and U6 were used as endogenous controls for mRNAs and miRNAs, respectively. All of the primer sequences are demonstrated in Additional file 2: Table S2.

### Wright‒Giemsa staining

Murine PB smears and BM cytospins were stained following standard protocols for morphological analysis [20]. Briefly, cells were collected on slides by cytospin (Shandon, Runcorn, United Kingdom) and stained by Wright‒Giemsa staining buffer for approximately 4 min. Cytospins were examined under a light microscope.

### Analysis of target genes

TargetScan (http://www.targetscan.org) was used to predict putative miRNA**‒**target pairs [21].

### DNA methylation detection

CpG islands at the miR-182 promoter were selected according to the following criteria: (1) > 200 bp in length; (2) cytosine and guanine content > 50%; (3) ≥ 60% of the observed/expected dinucleotides CpG. To determine the frequency of DNA hypermethylation at the miR-182 promoter, DNA was extracted by DNA Purification Kit (Vazyme Biotech) and treated with sodium bisulfite by EZ DNA Methylation™-GOLD Kit (ZYMO RESEARCH, Irvine, CA, USA). For methylation and unmethylation-specific PCR analysis (MSP and UMSP), the methylation-specific and unmethylation-specific primers were designed by MethPrimer software [22]. For bisulfite-sequencing analysis, sodium bisulfite-treated DNA was used as the template for PCR-mediated amplification of the CpG island at the miR-182 promoter. PCR products were subcloned and inserted into pUC18 vector for direct sequencing.

### MethylTarget™ assays

MethylTarget™ assays were performed by Genesky BioTech (Shanghai, China) [12, 23]. Briefly, we used an EZ DNA Methylation™-GOLD Kit (ZYMO RESEARCH) to transform all unmethylated cytosines to uracils. The samples with a bisulfite conversion rate < 98% were first filtered out. After the target CpG regions were amplified, separated, and purified by a gel extraction kit (Vazyme Biotech), a CpG island methylation assay was performed with an Illumina Hiseq/Miseq 2000 according to the manufacturer's protocol.

### BCR-ABL (P190)-induced B-ALL and Notch-induced murine T-ALL models

BM c-Kit^+^ cells were isolated from 8-week-old 182WT and 182KO mice and cultured in StemSpan SFEM (Stemcell Technologies) supplemented with murine SCF (50 ng/mL, PeproTech), IL-7 (10 ng/mL, PeproTech), IL-3 (10 ng/mL, PeproTech) overnight. Furthermore, c-Kit^+^ cells were retrovirally transduced with MSCV-green fluorescent protein (GFP)-internal ribosome entry site (IRES)-BCR-ABL (P190) or MSCV-IRES-GFP-IRES-Notch through two rounds of “spinoculation” as previously described [24]. Then, GFP^+^ cells were isolated by fluorescence-activated cell sorting 48 h after transduction and transplanted into lethally irradiated C57BL/6 J mice (Beijing Vital River Laboratory, Beijing, China). BM cells were isolated for secondary BMT when the mice demonstrated symptoms of death. All animal procedures and care were performed according to national and international policies and institutional guidelines of the ethics committee of the First Affiliated Hospital of Wenzhou Medical University.

### Other procedures

Chemical drugs, Western blot, cell proliferation, cell viability assay, apoptosis assays, retroviral and lentiviral production and transduction, colony formation, flow cytometry analysis, construction of plasmids, luciferase activity, H&E staining, transfection of scrambled miR-182 (SCR182), and RNA sequencing (RNA-seq) please see the Additional file 3.

### Statistical analysis

All the results were expressed as mean ± SD where applicable. The significance of the difference between groups was determined by Student's t-test. OS was estimated by the Kaplan–Meier method. The log-rank test was used to assess statistical significance. A *P* value of less than 0.05 was considered statistically significant. All statistical analyses were performed with Prism version 9.3.

## Results

### miR-182 expression is decreased in primary ALL cells because of miR-182 promoter hypermethylation

**Fig. 1:**
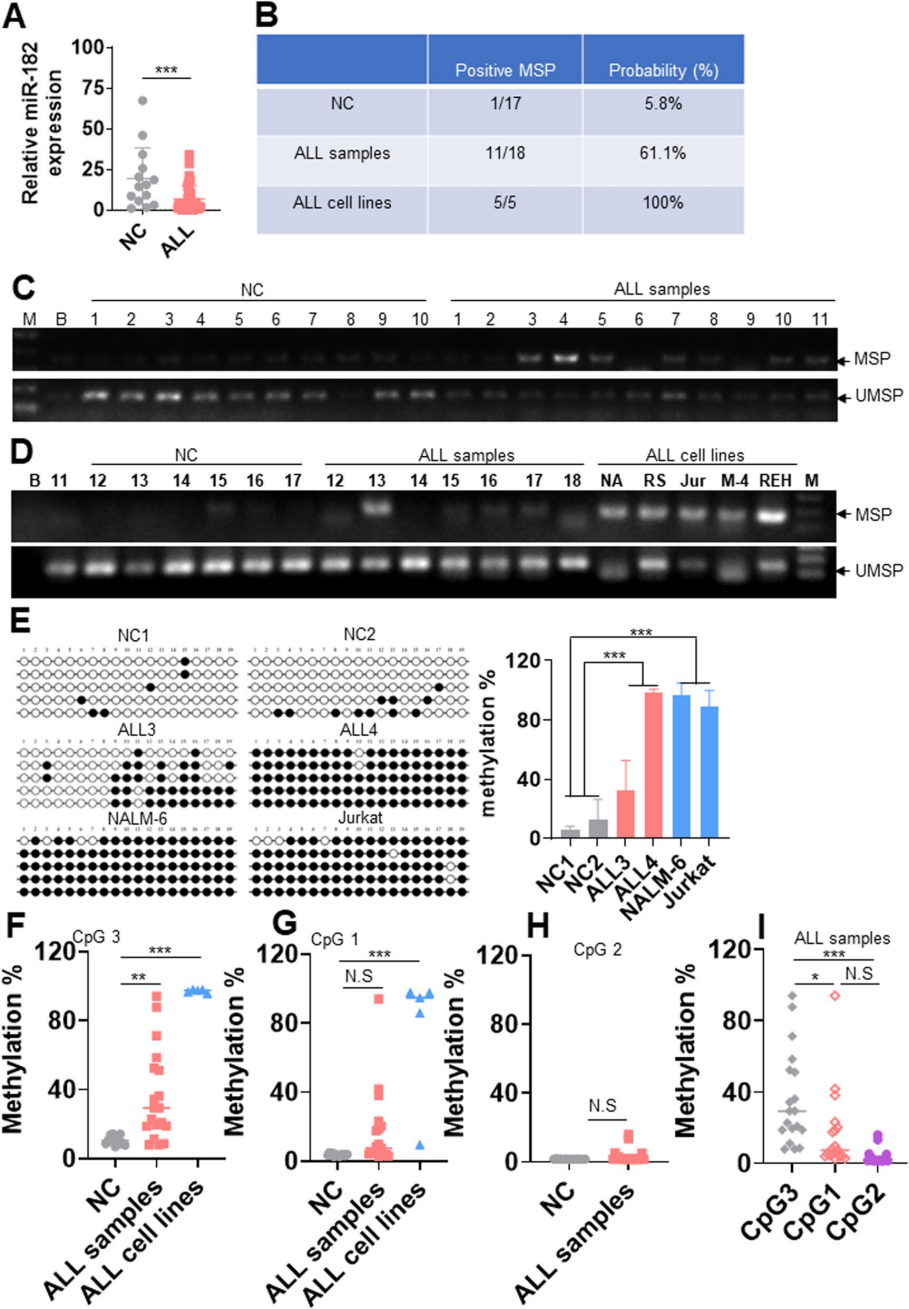
miR-182 expression is lower in B-ALL cells than in NCs because of miR-182 promoter hypermethylation. **A** Relative miR-182 levels were measured in 38 untreated B-ALL cells and 14 NCs. **B** Shown are positive MSP frequencies at CpG island 3 of the miR-182 promoter in 5 ALL cell lines, 18 B-ALL samples, and 17 NCs. **C** and **D** Analysis of MSP and UMSP at the miR-182 promoter in 5 ALL cell lines, 18 B-ALL samples, and 17 NCs. The Bands in the ‘MSP’ and ‘UMSP’ lanes are PCR products amplified with methylation-specific and unmethylation-specific primers, respectively. NA: NALM-6; RS: RS4;11; Jur: Jurkat; M-4: MOLT-4. **E** Bisulfite genomic sequencing was used to assess the methylation status of CpG island 3 in two NC samples, two B-ALL samples, NALM-6, and Jurkat cell lines. Five colonies were shown for each sample. Each row of the circle represents the sequence of an individual clone. The black and empty circles represent methylated and unmethylated CpG dinucleotides, respectively (Left). Shown is the statistical analysis of the percentage of methylation (Right). **F** and **G** A MethylTarget™ assay was performed to analyze the percentage of DNA methylation at CpG islands 3 and 1 in 14 NCs, 19 B-ALL samples, and 5 ALL cell lines, including NALM-6, REH, RS4;11, Jurkat, and MOLT-4 cells. **H** A MethylTarget™ assay was performed to analyze the different DNA methylation at CpG islands 2 in 14 NCs and 19 B-ALL samples. **I** The percentage of DNA methylation at CpG islands 3, 1, and 2 was analyzed in 19 B-ALL samples. *P < 0.05; **P < 0.01; ***P < 0.001. NS: Not significant

To explore the function of miR-182 in ALL cells, we first measured the miR-182 level in 38 untreated primary B-ALL samples (Additional file 1: Table S1) and 14 normal controls (NCs). miR-182 expression was significantly lower in primary ALL tissue samples than in NCs (Fig. 1A). However, no significant differences in miR-182 expression was found for gender, B-ALL with recurrent genetic abnormality, B-ALL with t(9;22) (q34;q11), or B-ALL not otherwise specified (Additional file 1: Table S1). We subsequently determined whether DNA hypermethylation mediates the low expression of miR-182 through the three CpG islands at the miR-182 promoter (Additional file 4: Fig. S1A and B) [12]. CpG island 3 is more heavily methylated than CpG island 1; while CpG island 2 is not methylated in AML cells [12]. Thus, to determine miR-182 promoter hypermethylation also occurs in ALL cells, we first used MSP and UMSP to rapidly assess CpG island 3 methylation level in 17 NCs and 18 primary untreated B-ALL cells because B-ALL accounts for more than 90% of adult ALL patients. MSP demonstrated that only 1 of 17 (5.8%) NC samples showed positive MPCR (Fig. 1B–D). However, 11 of 18 (61%) B-ALL samples and 5 of 5 (100%) ALL cell lines showed positive MPCR (Fig. 1B–D). In addition, bisulfite genomic sequencing of CpG island 3 revealed that methylation frequencies were almost 100% in two ALL cell lines (NALM-6 and Jurkat) and heavy methylation was detected in ALL3 and ALL4 (Fig. 1E). In contrast, methylation frequency was < 20% in two NC samples (Fig. 1E). Thus, the methylation frequencies were markedly higher in two ALL cell lines and two primary ALL samples than in the two NC samples (Fig. 1E). Finally, the MethylTarget™ assay was performed to quantitatively analyze the methylation frequency of CpG island 3 in 14 NC samples, 19 B-ALL samples, and 5 ALL cell lines. As expected, the average methylation frequencies of CpG island 3 in ALL cell lines and ALL samples were 95.9% and 35.8%, respectively, which were significantly higher than those in the NC samples (11.0%, Fig. 1F and Additional file 5: Fig. S2A). We then explored the methylation frequency of CpG island 1 in ALL samples, and found that although methylation frequency of CpG island 1 in ALL samples is higher than NC samples, no significant difference was found between them (Fig. 1G and Additional file 5: Fig. S2B). In consistent with the result in AML cells [12], CpG island 2 was not methylated (Fig. 1H and Additional file 6: Fig. S3A and B). Furthermore, the methylation frequency of CpG island 3 was markedly higher than that of CpG island 1 and 2 in primary ALL samples (Fig. 1I). The methylation frequencies at most individual CpG 3 sites were higher in the ALL samples than in the NC samples (Additional file 7: Fig. S4A). However, the methylation frequencies at most individual CpG 1 sites were the same in the ALL samples and NCs (Additional file 7: Fig. S4B). Overall, CpG island 3 represents the real methylation status in ALL samples and miR-182 promoter hypermethylation frequently occurs in ALL cells, which might lead to the decreased expression of miR-182 in ALL cells.

### Overexpression of miR-182 inhibits proliferation and induces apoptosis in ALL cells

**Fig. 2:**
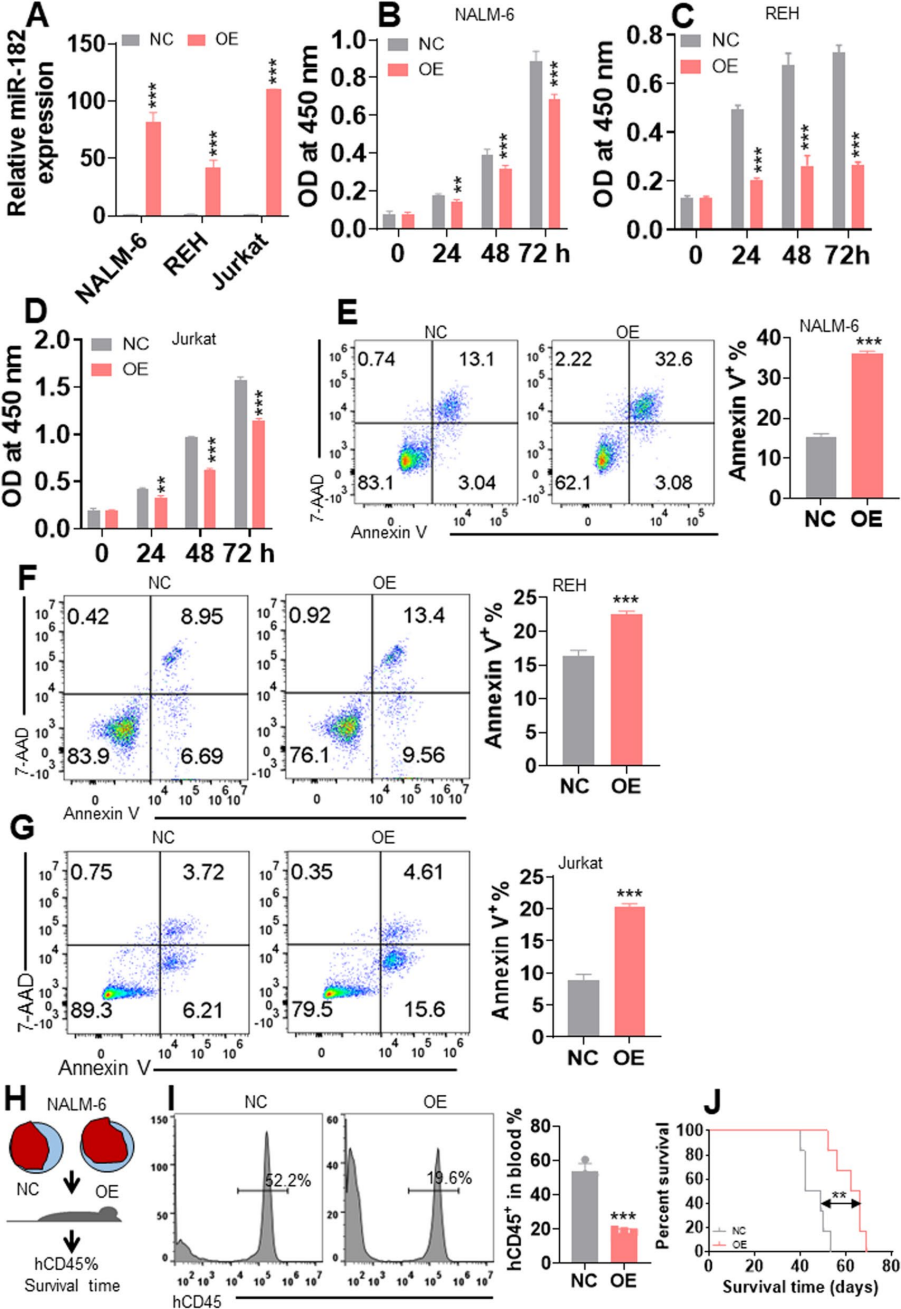
Overexpression of miR-182 inhibits proliferation and induces apoptosis in ALL cells. **A** miR-182 expression was measured in NALM-6, REH, and Jurkat cell lines transduced with blank vector (NC) or vector MSCV-miR-182 overexpressing miR-182 (OE). **B**–**D** CCK8 activity was measured in NALM-6, REH, and Jurkat cells transduced with NC or OE for the indicated days. **E**–**G** Apoptosis was measured in NALM-6, REH, and Jurkat cell lines overexpressing miR-182 or NC by Annexin V and 7-AAD staining. Representative flow cytometry plots (left) and statistical analysis of the percentage of Annexin V^+^ cells are shown. **H** A demonstration of the NALM-6-xenografted NSG mouse model. NALM-6 cells were transduced with 182OE or 182NC and transplanted into NSG mice. **I** hCD45^+^ cells in the PB of NALM-6-182OE or NALM-6-182NC-xenografted NSG mice were measured. Shown are the representative plots (left) and statistical analysis of the percentage of hCD45^+^ cells (right). **J** OS was determined in NALM-6-182OE and NALM-6-182NC-xenografted NSG mice. **P < 0.01; ***P < 0.001

Because miR-182 expression is lower in ALL cells than in NCs, we explored the anti-leukemia ability of miR-182 overexpression (182OE) in ALL cell lines, including NALM-6, REH, and Jurkat cells. The expression of miR-182 was increased 40–120-fold in 182OE-transduced cells compared with that in blank Vector (NC) (Fig. 2A). Furthermore, overexpression of miR-182 substantially decreased the proliferation, as determined by CCK8 assay (Fig. 2B–D), and induced apoptosis, as determined by annexin V/7-AAD assay (Fig. 2E–G). To further elucidate the role of miR-182 in ALL cells, equal amounts of NALM-6-182OE and NALM-6-182NC cells were transplanted in NSG mice, and hCD45^+^ percentage and OS were measured (Fig. 2H). 182OE-NALM-6 cells had a lower percentage of hCD45 (Fig. 2I) and extended OS compared with NALM-6-NC cells (Fig. 2J).

To determine whether overexpression of miR-182 specifically leads to the anti-ALL ability, ALL cells were transduced with a scrambled miR-182 sequence (SCR182) or blank control (Ctrl). Overexpression of SCR182 did not affect cell proliferation and apoptosis compared with Ctrl in NALM-6 and Jurkat cells (Additional file 8: Fig. S5A-D).

### Knockout of miR-182 (182KO) accelerates the development of murine BCR-ABL (P190)-transformed B-ALL

**Fig. 3:**
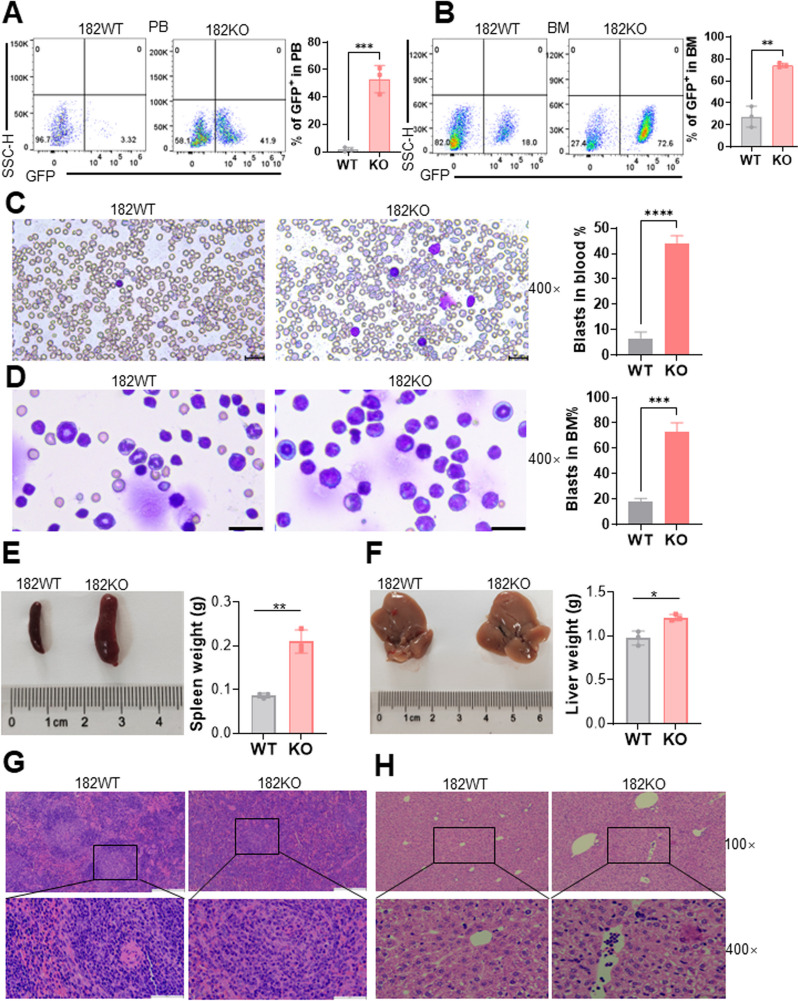
Depletion of miR-182 accelerates the development of murine BCR-ABL (P190)-transformed B-ALL. **A** and **B** The percentages of GFP^+^ cells were determined in PB (**A**, n = 3 for WT and KO mice) and BM cells (**B**, n = 3 for WT and KO mice) from 182WT and 182KO ALL mice. Shown are the representative plots (left) and statistical analysis of the percentages of GFP^+^ cells (right). (C and D) Wright-Giemsa staining for the PB (**C**) and BM (**D**) cells. Representative pictures (left) and statistical analysis of the percentages of blasts (right) are shown. The bar represents 20 μm. **E** and **F** Spleen (**E**) and liver (**F**) weights were measured in 182WT and 182KO ALL mice (n = 3 for WT and KO mice). Shown are the representative pictures (left) and statistical analysis of the spleen and liver weights (right). **G** and **H** HE staining of spleen (**G**) and liver (**H**) tissues from 182WT and 182KO ALL mice. The bar represents 50 μm.*P < 0.05; **P < 0.01; ***P < 0.001; ****P < 0.0001

To explore the function of miR-182 in vivo, we used 182KO mice for further study [25]. Genotyping demonstrated that 182KO mice were successfully produced (Additional file 9: Fig. S6A). BM c-Kit^+^ cells were isolated from miR-182 knockout (182KO) and WT mice to construct BCR-ABL (P190)-transformed B-ALL model (Additional file 9: Fig. S6B). When the mice were severely ill, including hyperleukocytosis, paralysis, or slow movement, mice were sacrificed, and BM cells were isolated for secondary BMT. GFP^+^ cells were isolated from 182WT and 182KO B-ALL mice to measure the miR-182 expression, which was fully depleted in 182KO B-ALL cells compared with 182WT B-ALL cells (Additional file 9: Fig. S6C). In addition, immunophenotyping analysis demonstrated that GFP^+^ ALL cells only express B220, but not CD3, Mac-1, and Gr-1 (Additional file 9: Fig. S6D), suggesting that B-ALL model was successful. The percentage of GFP^+^ cells representing leukemia cells was significantly higher in 182KO B-ALL cells compared to 182WT B-ALL cells in the peripheral blood (PB, Fig. 3A) and BM cells (Fig. 3B). In addition, the Wright-Giemsa staining demonstrated a higher percentage of ALL blasts in the PB (Fig. 3C) and BM cells (Fig. 3D) in 182KO than 182WT B-ALL cells. These results demonstrated a higher B-ALL burden in 182KO than in 182WT B-ALL cells. We subsequently determined whether 182KO B-ALL cells have higher infiltration in the spleen and liver. The spleen and liver weights were markedly higher in 182KO B-ALL mice than in 182WT B-ALL mice (Fig. 3E and F). Additionally, 182KO B-ALL mice had a more extensive leukemic infiltration in the spleen (Fig. 3G) and liver tissues (Fig. 3H) than in 182WT B-ALL mice. Collectively, 182KO B-ALL cells have much higher leukemogenic activities than 182WT B-ALL cells.

### Murine 182KO B-ALL cells are more enriched in LIC than 182WT B-ALL cells are

**Fig. 4.**
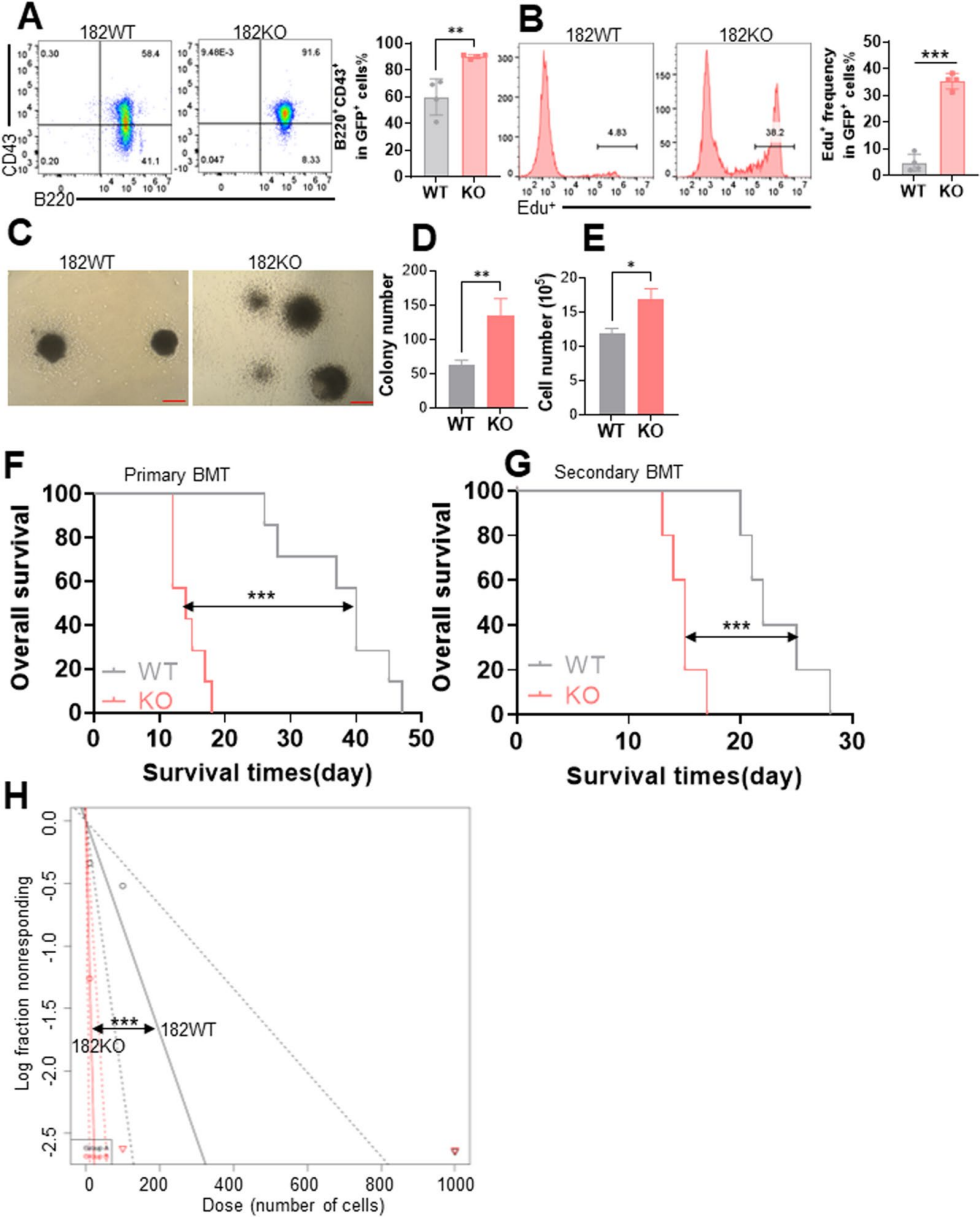
182KO ALL cells are enriched more LIC (B220^+^CD43^+^) cells. **A** The percentage of LIC was measured in BM GFP^+^ cells from 182WT and 182KO ALL mice. Representative flow plots (left) and statistical analysis of the percentage of LIC (right) are shown. **B** Edu staining for BM GFP^+^ cells from 182WT and 182KO ALL mice. Shown are the representative flow plots (left) and statistical analysis of the percentage of Edu^+^ cells (right). **C**–**E** BM GFP^+^ cells were sorted from 182WT and 182KO ALL mice and plated on murine methylcellulose medium (8000/dish). Colony number (D) was counted after ten days of culture, and total cell number (**E**) was measured after colonies were washed. The Bar represent 10 μm. **F** and **G** OS was counted in primary (n = 7 for WT and KO mice) and secondary BMT (n = 5 for WT and KO mice). **H** Limiting dilution assay of BM GFP^+^ cells from secondary BMT leukemic mice with 182WT (n = 7) and 182KO (n = 7). The frequency of LSC and *P* value were determined via L-calc software. *P < 0.05; **P < 0.01; ***P < 0.001

Because murine LIC (B220^+^CD43^+^) affects the leukemogenesis of B-ALL [26, 27], we assessed whether 182KO B-ALL cells have a higher percentage of LIC than 182WT B-ALL cells. As demonstrated in Fig. 4A, the immunophenotypic LIC in the BM of the recipients receiving 182KO B-ALL cells was much higher than that in the mice receiving 182WT B-ALL cells. Furthermore, 182KO B-ALL cells have about tenfold higher percentage of Edu^+^ cells (Fig. 4B) than 182WT B-ALL cells, suggesting that 182KO B-ALL cells have higher proliferation activity than 182WT B-ALL cells. In addition, the number of colony formation representing functional self-renewal ability was counted. An approximately 2.0-fold increase in colony numbers (Fig. 4C and D) and a 1.5-fold increase in total cell numbers (Fig. 4E) were found in 182KO B-ALL cells compared with 182WT B-ALL cells. Furthermore, OS was significantly shorter in primary and secondary BMT of 182KO B-ALL mice than 182WT B-ALL mice (Fig. 4F and G). Finally, we measured the functional LSC frequency by limiting dilution analysis (LDA) [28]. The estimated LSC frequency was increased by approximately 8.5-fold in 182KO B-ALL mice than that in 182WT AML mice by LDA (Fig. 4H and Additional file 10: Table S3).

To explore the pathways associated with the dysregulation of 182KO and 182WT B-ALL cells, BM GFP^+^ cells from 182KO and 182WT B-ALL mice were isolated for RNA-seq. Pathway analysis demonstrated that Hippo signaling and pathway in cancer were enriched in 182KO B-ALL cells compared to 182WT B-ALL cells (Additional file 11: Fig. S7A and S7B).

### 182KO accelerates the development of murine Notch-transformed T-ALL model

To further investigate the potential role of miR-182 in T-ALL cells, BM c-Kit^+^ cells were isolated from 182KO and 182WT mice to construct Notch-transformed T-ALL model. BM GFP^+^ cells wholly expressed CD3 but not CD19, Mac-1, and Gr-1 from 182KO and 182WT T-ALL mice (Additional file 12: Fig. S8A), suggesting that T-ALL model was successful [29]. Because leukemic infiltration in thymic tissue is an important characteristic in T-ALL cells, we examined the thymic tissue by HE staining. A higher infiltration of leukemic cells was found in the thymic tissue from 182KO T-ALL mice compared with 182WT T-ALL mice (Additional file 12: Fig. S8B). Finally, the OS was significantly shorter in 182KO T-ALL mice compared with 182WT T-ALL mice (Additional file 12: Fig. S8C).

### PBX3 and BCL2 are two direct targets of miR-182

**Fig. 5:**
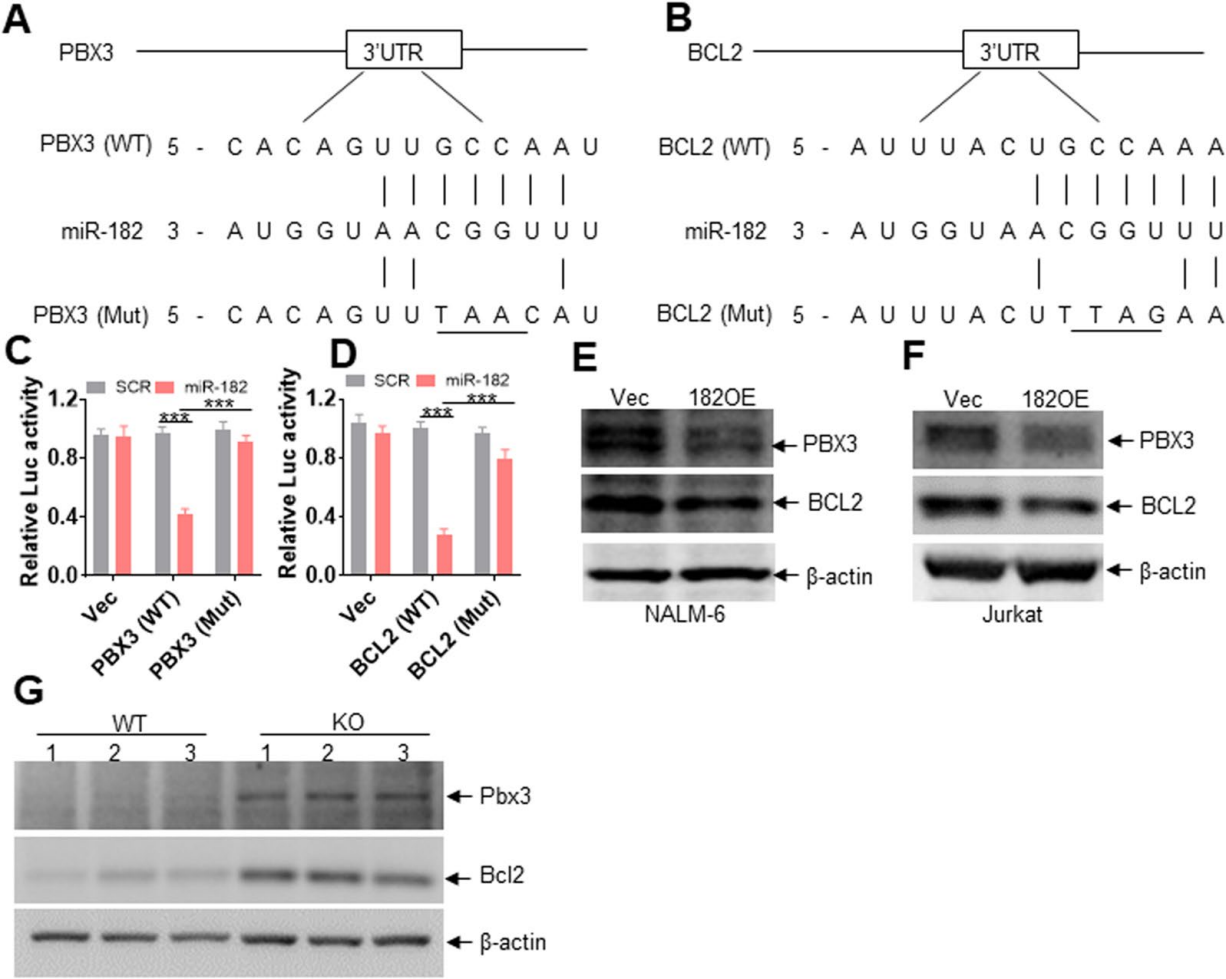
PBX3 and BCL2 are two direct targets of miR-182. **A** and **B** An illustration of the potential binding sites of miR-182 in the 3'-UTR of *PBX3* (**A**) and *BCL2* (**B**). **C** HeLa cells were transfected with sicheck-2-PBX3-3'-UTR (WT) or sicheck-2-PBX3-3'-UTR (Mut) for 24 h, as well as with miR-182 mimics, or scramble (SCR) for another 24 h. Cell lysates were collected, and Firefly and Renilla luciferase activities were both measured. **D** HeLa cells were transfected with sicheck-2-BCL2-3'-UTR (WT) or sicheck-2- BCL2-3'-UTR (Mut) for 24 h, as well as miR-182 mimics, or SCR for another 24 h. Firefly and Renilla luciferase activities were both measured in the collected cell lysates. The Firefly/Renilla luciferase activities are shown. **E** and **F** The protein expression levels of PBX3 and BCL2 were measured in NALM-6 and Jurkat cells transduced with 182OE or Vec. **G** The protein expression levels of Pbx3 and Bcl2 were measured in BM GFP^+^ cells from murine 182WT and 182KO B-ALL model. ***P < 0.001

To further explore the potential targets of miR-182 in ALL cells, we first searched for the possible targets of miR-182 via TargetScan (http://www.targetscan.org). Pre-B-cell leukemia transcription factor 3 (PBX3) and BCL2, two important oncogenes associated with the proliferation, apoptosis, and stemness of leukemic cells [30, 31], were ultimately selected for the following experiments. As shown in Fig. 5A and B, conserved regions in 3-'UTR of *PBX3* and *BCL2* mRNA may bind to miR-182. In addition, the binding sequences of miR-182 at 3-'UTR of *PBX3* and *BCL2* were inserted into a vector carrying Luc gene to measure Luc activity. Overexpression of miR-182 and the regular binding sites of miR-182 reduced Luc activity by more than twofold (Fig. 5C and D). In contrast, overexpression of miR-182 and the mutation of the binding sites of miR-182 rescued the decrease in Luc activity induced by miR-182 (Fig. 5C and D). We subsequently measured the protein expressions of PBX3 and BCL2 in miR-182-overexpressing or miR-NC-overexpressing ALL cells. Overexpression of miR-182 markedly decreased the protein expressions of PBX3 and BCL2 than overexpression of miR-NC in NALM-6 and Jurkat cells (Fig. 5E and F). Consistent with the finding in human cells, murine 182KO B-ALL cells had higher protein expressions of Bcl2 and Pbx3 than 182WT B-ALL cells (Fig. 5G).

Because miRNAs degrade or repress the translation of target mRNAs, we subsequently measured *PBX3* and *BCL2* transcript levels in ALL cells overexpressing miR-182. As indicated in Additional file 13: Fig. S9A and B, overexpression of miR-182 did not substantially affect *PBX3* and *BCL2* transcript levels in NALM-6 and Jurkat cells. In addition, *Pbx3* and* Bcl2* transcript levels were similar between 182KO and 182WT murine B-ALL cells according to RNA-seq analysis (Additional file 13: Fig. S9C), suggesting that miR-182 might repress the translation of *PBX3* and *BCL2*.

### HMAs have anti-ALL effects via miR-182-PBX3/BCL2 axis

**Fig. 6:**
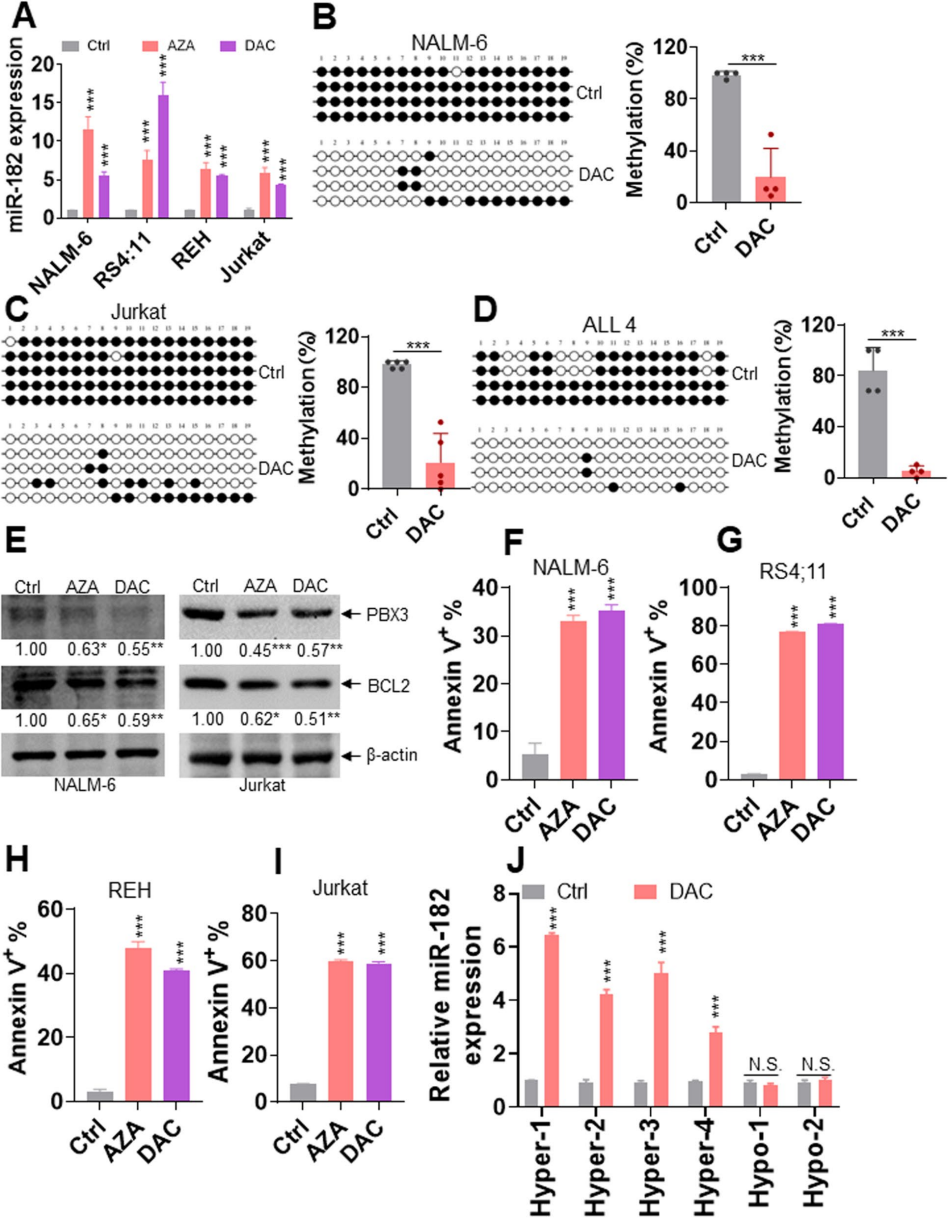
HMAs have notable anti-ALL activity through the miR-182-PBX3/BCL2 axis in ALL cells. **A** miR-182 levels were measured in NALM-6, REH, RS4;11, and Jurkat cells treated with or without AZA (5 μM) and DAC (5 μM) for four days. **B** and **C** The CpG 3 methylation frequency was analyzed by bisulfite genomic sequencing in NALM-6 and Jurkat cells treated with or without DAC (5 μM) for four days. Each row of circles represents the sequence of an individual clone. The black and empty circles represent methylated and unmethylated CpG dinucleotides, respectively (Left). Shown are the statistical analysis of the percentage of methylation (Right). **D** The frequency of methylation at CpG 3 was analyzed by bisulfite genomic sequencing in one primary ALL sample treated with or without DAC (5 μM) for two days. Shown is the statistical analysis of the percentage of methylation (Right). **E** The protein expression levels of PBX3 and BCL2 were measured in NALM-6 and Jurkat cells incubated with or without AZA (5 μM) and DAC (5 μM) for four days. **F**–**I** Apoptosis was measured in NALM-6, RS4;11, REH, and Jurkat cells treated with or without AZA (5 μM) and DAC (5 μM) for four days. **J** miR-182 levels were measured in four primary ALL samples with miR-182 promoter hypermethylation and two with hypomethylation that were treated with DAC (5 μM) for two days. *P < 0.05; **P < 0.01; ***P < 0.001. NS: Not significant

Our results demonstrated that miR-182 expression was lower in ALL cells than in normal cells because of miR-182 promoter hypermethylation. Furthermore, miR-182 decreased the expressions of PBX3/BCL2 protein. Therefore, we determined whether AZA and DAC have anti-ALL effects through the miR-182-PBX3/BCL2 axis. ALL cells were treated with AZA and DAC for four days, after which miR-182 expression was first measured. As expected, AZA and DAC increased the expression of miR-182 in all four ALL cell lines (Fig. 6A). We wanted to explore whether the increased expression of miR-182 by DAC treatment was caused by the decreased frequency of methylation at the miR-182 promoter. DAC treatment reduced the methylation frequency of CpG 3 from 98 to 19% in NALM-6 cells (Fig. 6B) and from 97 to 21% in Jurkat cells (Fig. 6C) according to bisulfite genomic sequencing. We also measured the methylation frequency in one ALL4 sample treated with or without DAC in vitro. DAC treatment reduced the methylation frequency from 84 to 5% in ALL4 (Fig. 6D). In addition, AZA and DAC reduced the protein expressions of PBX3/BCL2 in NALM-6 and Jurkat cells, respectively (Fig. 6E). Furthermore, AZA and DAC substantially induced apoptosis in four ALL cell lines (Fig. 6F–I and Additional file 14: Fig. S10A–D). We then explored whether DAC regulates the expression of miR-182 in primary ALL cells. DAC increased the miR-182 expression in four primary ALL samples with hypermethylation, but did not regulate the miR-182 expression in two primary ALL samples with hypomethylation (Fig. 6J). Overall, methylation of CpG 3 might mediate the silencing of miR-182 in ALL cells and DAC increases the miR-182 level by reducing the methylation of CpG 3 at the miR-182 promoter.

### DAC cooperates with Ven to reduce the viability of ALL samples with miR-182 promoter hypermethylation

**Fig. 7:**
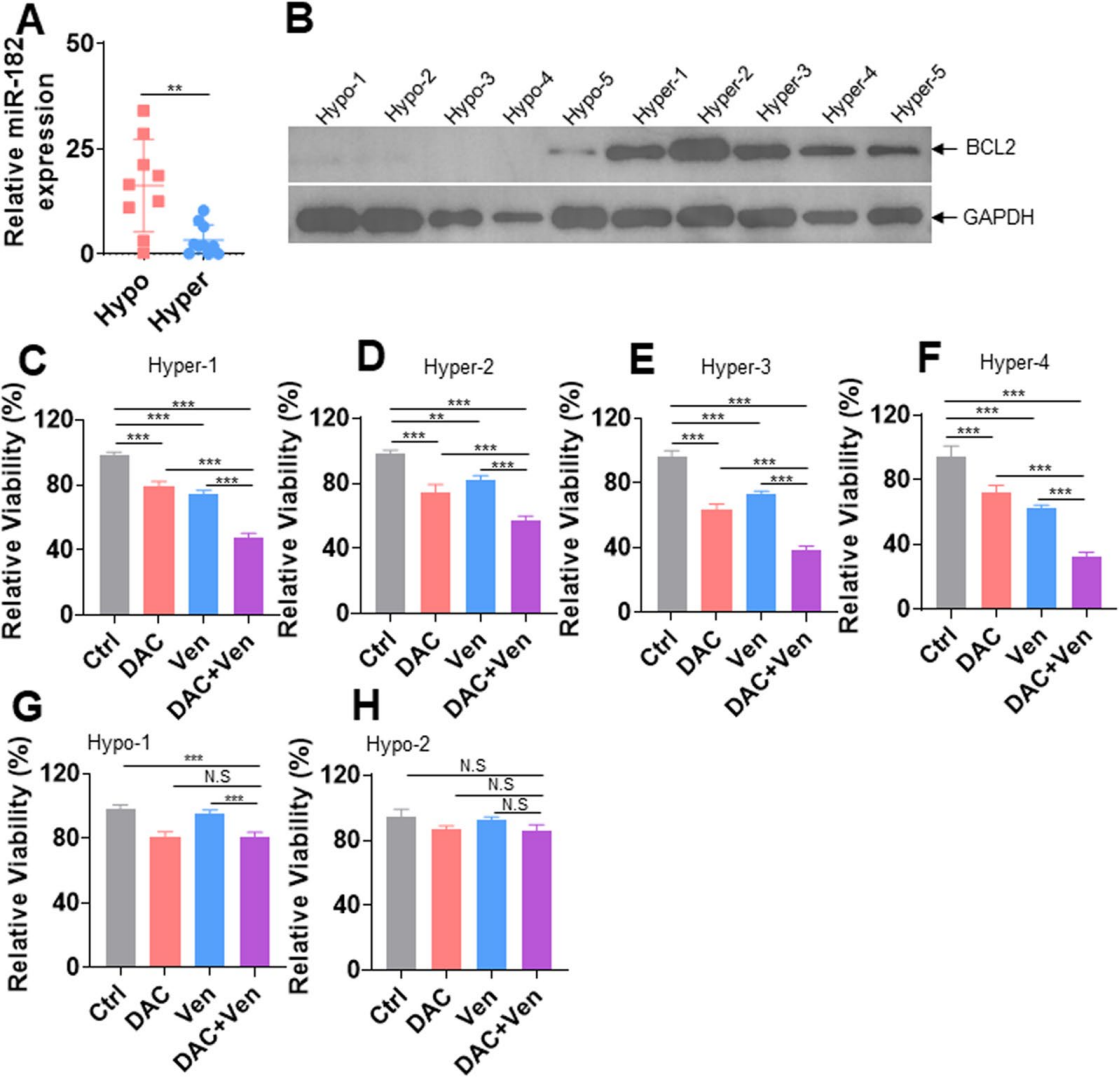
miR-182 promoter methylation is a predictive marker for DAC + Ven treatment. **A** miR-182 expression was analyzed in ALL cells with DNA hypermethylation or hypomethylation at the miR-182 promoter. **B** BCL2 protein levels were measured in five ALL samples with miR-182 promoter hypomethylation (hypo) and five ALL samples with hypermethylation (hyper). **C**–**H** Cell viability was measured in four ALL samples with miR-182 hypermethylation (**C**–**F**) and two ALL samples with hypomethylation (**G** and **H**) treated with Ven (0.1 μM) + DAC (5.0 μM) or either of them for 24 h. **P < 0.01; ***P < 0.001. N.S: not significant

Although the average methylation frequency was higher in ALL samples than in NCs, some ALL samples still presented low methylation (Fig. 1F and Additional file 5: Fig. S2A). We first explored whether DNA hypermethylation was associated with low expression of miR-182. Because the median methylation frequency of 19 B-ALL samples was 29.3%, we arbitrarily set the cutoff to 29%. miR-182 expression in ALL samples with DNA hypermethylation (> 29%) was significantly lower than those with DNA hypomethylation (< 29%) (Fig. 7A). Our data have demonstrated that BCL2 is a direct target of miR-182. We subsequently determined whether miR-182 promoter hypermethylation-mediated low expression of miR-182 was associated with high levels of BCL2 protein in ALL samples. Five ALL samples with hypermethylation and five with hypomethylation were collected to measure the protein level of BCL2. As expected, ALL samples with miR-182 promoter hypermethylation had higher BCL2 protein expressions than did those with hypomethylation (Fig. 7B). As reported that Ven is sensitive to the leukemic cells expressing high levels of the BCL2 protein [32], we explored whether DAC has synergistic activity with Ven in ALL cells with the miR-182 promoter hypermethylation. Six ALL samples (four hypermethylation and two hypomethylation) were treated with DAC (5.0 μM) + Ven (0.1 μM) or either of them for 24 h, and cell viability was measured. DAC + Ven synergistically reduced cell viability than either of them in four primary ALL samples (Fig. 7C–F) with miR-182 promoter hypermethylation, but not in two primary ALL samples with miR-182 promoter hypomethylation (Fig. 7G and H).

## Discussion

Our study revealed that miR-182 expression is silenced in ALL cells because of promoter hypermethylation. 182KO accelerates the development of murine B-and T-ALL models compared with 182WT. Furthermore, overexpression of miR-182 inhibits proliferation and induces apoptosis via targeting PBX3 and BCL2, suggesting that miR-182 acts as a tumor suppressor gene in ALL cells. DAC treatment increases the expression of miR-182 by reducing methylation level, followed by downregulation of PBX3/BCL2. Thus, DAC exerts its anti-ALL effects by regulating miR-182-PBX3/BCL2 axis. Most importantly, DAC cooperates with Ven to reduce viability in primary ALL samples with miR-182 promoter hypermethylation but not hypomethylation. In conclusion, ALL cells with miR-182 promoter hypermethylation, which present low expression of miR-182 and high protein level of BCL2, are sensitive to DAC + Ven cotreatment, suggesting that DAC + Ven has further clinical application for ALL patients with miR-182 promoter hypermethylation.

Although most studies have demonstrated that miR-182 functions as tumor suppressor miRNA in different tumors [9–11, 33], several reports indicate that miR-182 acts as an oncogenic miRNA [7, 34]. We used miR-182 KO and WT cells to construct ALL models and found that miR-182 depletion rapidly accelerates disease development via enriching more LIC compared with miR-182 WT cells. Additionally, overexpression of miR-182 inhibits proliferation and induces apoptosis in ALL cell lines. Thus, our results confirm that miR-182 acts as a tumor suppressor miRNA in ALL cells, which is consistent with our finding that miR-182 inhibits cell growth in AML cells [12]. Because miRNAs can directly or indirectly regulate multiple targets with potential counteracting roles, the cell context-dependent balance in miR-182 targets may ultimately determine the biological functions of ALL cells.

Multiple reports have indicated that hypermethylation at the DNA promoter leads to the silencing of correlated genes [35, 36]. Although Xu et al*.* demonstrated that hypermethylation of the miR-182 promoter frequently occurs in renal cell carcinoma but not in adjacent normal tissues [25], the exact methylation sites correlated with miR-182 expression are unknown in ALL cells. Here, we thoroughly investigated methylation sites at miR-182 promoter and identified three potential CpG islands. Among the three CpG islands, CpG island 3 is more heavily methylated than CpG island 1. By contrast, CpG island 2 is not methylated in ALL cells. These results are consistent with our finding that the frequency methylation at CpG island 3 is higher than that at CpG island 1 in AML cells [12]. Our results demonstrated that methylation frequency of CpG island 3 is significantly higher at ALL cell lines than primary ALL cells from patients, probably because cell lines generally have different DNA methylation profiles as compared to primary material. Klco et al*.* demonstrated that although DAC treatment induces hypomethylation at highly methylated CpGs, it cannot substantially modulate transcriptional activity [37]. However, we found that DAC treatment substantially reduces the methylation frequency of CpG island 3 in vitro, followed by the increased expression of miR-182. These results strongly suggest that CpG island 3 might be the real methylation site mediating the silencing of miR-182 in ALL cells. In conclusion, in this study, we first used MPCR, sodium bisulfite sequencing, and MethylTarget™ assay to elucidate that miR-182 promoter hypermethylation frequently occurs in ALL cells, and CpG island 3 represents the real methylated site.

HMAs alone or with other drugs have been widely applied for the treatment of myelodysplastic syndrome (MDS) and AML [38, 39]. Moreover, HMAs + Ven cotreatment substantially improves the OS in elderly AML patients [18]. However, only a few studies indicated that HMAs alone or HMAs + Ven cotreatment has the anti-ALL ability and apply in the clinical try for ALL patients [40–42]. For example, Cheung et al. reported that HMA alone or HMAs + Ven are highly effective in infant *KMT2A*-rearranged ALL cells, which exhibit high BCL2 expression [40]. Our analysis indicated that HMAs markedly induce apoptosis in ALL cell lines, which all present miR-182 promoter hypermethylation. Thus, HMAs induce apoptosis in ALL cell lines with the miR-182 promoter hypermethylation by increasing miR-182 expression, followed by the degradation of the BCL2 protein. Most importantly, ALL samples with miR-182 promoter hypermethylation and high BCL2 protein level are more sensitive to DAC + Ven cotreatment than those with miR-182 promoter hypomethylation. Therefore, methylation status at the miR-182 promoter might be a predictive marker for HMAs + Ven in ALL patients.

BCL2 is vital for the survival and self-renewal of LSC, and inhibiting BCL2 selectively eradicates quiescent LSC in AML cells [31]. However, whether BCL2 can be a target in ALL patients is not determined. Peirs et al*.* reported that Ven is highly sensitive to some immature, TLX3- or HOXA-positive primary T-ALL cells, which have high levels of BCL2 protein [43]. However, the response of more differentiated T-ALL cells expressing low levels of BCL2 protein is poor [43]. Additionally, Ven treatment achieves a durable and complete response in high-risk relapsed and refractory adult B-ALL patients with high expression of BCL2/BCL2L1 [44]. Furthermore, Sarah et al*.* reported that freshly isolated ALL blasts expressing high levels of BCL2 exhibit high sensitivity to Ven [45]. These reports confirm that leukemic cells with high levels of BCL2 are sensitive to Ven treatment, but leukemic cells with lost expression of BCL2 protein are resistance to Ven treatment [32]. Consistent with these reports, ALL cells with miR-182 promoter hypermethylation and high levels of BCL2 protein are sensitive to DAC + Ven cotreatment in vitro. Our results might provide a quick method to select ALL patients by measuring miR-182 promoter methylation to make treatment decisions for the use of DAC + Ven. However, our samples are small and more additional ALL samples are required to investigate the efficiency of DAC + Ven in clinical try.

Consistent with the finding that miR-182 directly targets BCL2 in AML cells [12], we confirmed that BCL2 is a direct target of miR-182 in ALL cells. Additionally, several reports indicate that overexpression of miR-182 inhibits the expression of BCL2 [33, 46]. However, in addition to miR-182, BCL2 is also regulated by other miRNAs such as miR-15a/16-1 [47], suggesting the presence of a complicated network of miRNA-BCL2 axis. Although DAC downregulates BCL2 protein expression through increasing miR-182 expression, whether DAC regulates BCL2 expression through other miRNAs is still unknown.

PBX3 increases the DNA-binding or transcriptional activity of HOX proteins, and PBX3 cooperates with MEIS1 to rapidly transform hematopoietic cells into LSC [48, 49]. Targeting PBX3/MEIS1 interaction is a feasible strategy for treating MLL-rearranged leukemia cells overexpressing PBX3. In addition, PBX3 enhances EMT and maintains an aggressive phenotype in prostate cancer [50, 51]. These reports demonstrated that overexpression of PBX3 maintains a malignant phenotype and may provide a therapeutic target. Our results demonstrated that overexpression of miR-182 reduces PBX3 protein expressions in human ALL cells. In contrast, depletion of murine miR-182 increases Pbx3 protein level compared with depletion of negative control. In addition, overexpression of miR-182 reduces the luciferase activity of 3'-UTR of PBX3. These results suggest that PBX3 is a direct target of miR-182. Most importantly, HMAs treatment reduces PBX3 protein level through increasing miR-182 expression. Our results suggest a new mechanism by which HMAs regulate the miR-182-PBX3 axis.

## Conclusions

Here, we found that low expression of miR-182 caused by DNA hypermethylation facilitates the malignant phenotype of ALL through targeting PBX3/BCL2. HMAs present cytotoxic effects on ALL cells through the miR-182-PBX3/BCL2 axis. Furthermore, HMAs + Ven cotreatment should be applied in the clinical try of ALL patients with miR-182 promoter hypermethylation, which might be a predictive biomarker for HMAs + Ven cotreatment.

### Supplementary Information

**Additional file 1.** The clinical characteristics of 38 B-ALL patients.

**Additional file 2.** The sequences of primers for qRT-PCR and construction of plasmids.

**Additional file 3.** Supplemental material and methods.

**Additional file 4: Fig. S1.** The detailed three CpG islands at miR-182 promoter. (A) The regions indicating the CpG islands at the miR-182 promoter. TSS: transcription start site. (B) The detailed base information of three CpG islands

**Additional file 5: Fig. S2.** The methylation frequency of CpG island 3 and 1 at the miR-182 promoter by MethylTargetTM assay. (A and B) The detail methylation of CpG island 3 and 1 in 14 NCs, 19 primary ALL blasts, and 5 ALL cell lines

**Additional file 6: Fig. S3.** The methylation frequency of CpG island 2 at the miR-182 promoter by MethylTargetTM assay. (A and B) The detailed methylation information of CpG island 2 in 14 NCs and 19 primary ALL blasts

**Additional file 7: Fig. S4** Individual site analysis of methylation frequency in CpG island 3 and 1. (A and B) Individual site analysis of methylation frequency in CpG island 3 (A) and 1 (B) was performed in 14 NCs and 19 primary ALL blasts

**Additional file 8: Fig S5.** Overexpression of a scrambled miR-182 (SCR182) does not affect cell proliferation and apoptosis in ALL cells. (A and B) CCK8 activity was measured in NALM-6 and Jurkat cells transfected with SCR182 or blank control (Ctrl) for the indicated days. (C and D) Apoptosis was measured in NALM-6 and Jurkat cells transfected with SCR182 or Ctrl for 48 h by Annexin V and 7-AAD staining. Representative flow cytometry plots (left) and statistical analysis of the percentage of Annexin V+ cells (right) are shown. N.S: not significant

**Additional file 9: Fig S6.** Genotype of WT mice (182WT) and miR-182-knockout (182KO) mice and schedule for BCR-ABL (P190)-induced B-ALL mouse model. (A) Genotyping PCR for 182WT and 182KO mice. (B) Schedule for BCR-ABL (P190)-induced B-ALL mouse model. (C) Murine miR-182 expression was measured by qRT-PCR in BM GFP+ cells from 182WT B-ALL mice and 182KO B-ALL mice. (D) CD3, B220, Mac-1, and Gr-1 expressions were measured in BM GFP+ cells from primary 182WT and 182KO B-ALL mice by flow cytometry. Shown are the representative flow cytometry plots (left) and statistical analysis of T lymphocyte (CD3+), B lymphocyte (B220+), monocyte and granulocyte (Mac-1+ or Gr-1+) (Right). ***P<0.001; N.S: not significant

**Additional file 10.** Limiting dilution assay of BCR-ABL (P190)-transduced 182WT and 182KO B-ALL mice.

**Additional file 11: Fig S7.** RNA-seq for BM GFP+ cells from 182WT B-ALL mice and 182KO B-ALL mice. (A and B) Pathway analysis demonstrated that Hippo signaling, including Wnt and Yap-1, and pathway in cancer, including Ccnd2 and Fzd7, were enriched in 182KO B-ALL cells compared with 182WT B-ALL cells

**Additional file 12: Fig S8.** Depletion of miR-182 accelerates the development of murine Notch-transformed T-ALL model. (A) CD3, B220, Mac-1, and Gr-1 expressions were measured in BM GFP+ cells from 182WT and 182KO T-ALL mice. Shown are the representative flow cytometry plots (left) and statistical analysis of T lymphocyte (CD3+), B lymphocyte (B220+), monocyte, and granulocyte (Mac-1+ and Gr-1+). (B) HE staining of thymus tissue from 182WT and 182KO T-ALL mice. Bar for 10× is 200 μm and for 40× is 20 μm. (C) OS was analyzed in primary BMT (N= 6 for WT and KO). *P<0.05; N.S: not significant

**Additional file 13: Fig S9.** Overexpression of miR-182 does not significantly affect PBX3 and BCL2 transcript levels. (A and B) The transcript levels of PBX3 and BCL2 were measured in NALM-6 and Jurkat cells transduced with MSCV-miR-182 overexpressing miR-182 (182OE) or blank vector (Vec). (C) The relative Pbx3 and Bcl2 transcript levels were analyzed in BM GFP+ cells from murine 182WT and 182KO B-ALL model according to RNA-seq analysis. N.S: not significant

**Additional file 14: Fig S10.** HMAs induce apoptosis in ALL cells. (A-D) Apoptosis was measured by Annexin V/7-AAD staining in four ALL cell lines treated with or without DAC (5 μM) or AZA (5 μM) for four days. Representative flow cytometry plots were shown

## Data Availability

The datasets used and/or analyzed during the current study are available from the corresponding author on reasonable request.

## Abbreviations

ALL: Acute lymphocyte leukemia
AZA: Azacitidine
DAC: Decitabine
BM: Bone marrow
FBS: Fetal bovine serum
HMA: Hypomethylating agent
HSPC: Hematopoietic stem and progenitor cell
miRNA: MicroRNA
LICs: Leukemia-initiating cells
CpG: Cytosine-phosphate-guanine
CR: Complete remission
MNCs: Mononuclear cells
GFP: Green fluorescent protein
OS: Overall survival
Venetoclax: Ven
PBX3: Pre-B-cell leukemia transcription factor 3
MDS: Myelodysplastic syndrome
