# Supplementary material for: Low expression of miR-182 caused by DNA hypermethylation accelerates acute lymphocyte leukemia development by targeting PBX3 and BCL2: miR-182 promoter methylation is a predictive marker for hypomethylation agents + BCL2 inhibitor venetoclax
Source: Clin Epigenetics. 2024 Mar 26;16:48. doi: 10.1186/s13148-024-01658-2 (PMC10964616; doi:10.1186/s13148-024-01658-2)
Supplement: Supplementary file 1 — Additional file 1. The clinical characteristics of 38 B-ALL patients. [file 13148_2024_1658_MOESM1_ESM.docx]

**Table S1: the clinical characteristics of 38 B-ALL patients**

| Characteristic | ALL patients N (%) | *P* |
| --- | --- | --- |
| Overall | 38 |  |
| Gender |  |  |
| Male | 21(55.3) | 0.25 |
| Female | 17(44.7) |  |
| Age (range) | 56(19-78) |  |
| B-ALL with recurrent genetic abnormality | 17(44.7) | 0.15 |
| B-ALL with t(9;22)(q34;q11)[BCR-ABL1] | 8(21.0) |  |
| B-ALL with t(11q23)[MLL rearranged] | 3(7.9) |  |
| B-ALL with t(12;21)(p13;q22)[ETV6-RUNX1] | 2(5.2) |  |
| B-ALL with t(1;19)(q23;p13.3) [TCF3-PBX1] | 1(2.6) |  |
| B-ALL with translocations involving tyrosine or cytokine receptors (BCR-ABL1-like ALL) | 3(7.9) |  |
| B-ALL not otherwise specified | 21(55.3) |  |
