# Supplementary material for: Low expression of miR-182 caused by DNA hypermethylation accelerates acute lymphocyte leukemia development by targeting PBX3 and BCL2: miR-182 promoter methylation is a predictive marker for hypomethylation agents + BCL2 inhibitor venetoclax
Source: Clin Epigenetics. 2024 Mar 26;16:48. doi: 10.1186/s13148-024-01658-2 (PMC10964616; doi:10.1186/s13148-024-01658-2)
Supplement: Supplementary file 2 — Additional file 2. The sequences of primers for qRT-PCR and construction of plasmids. [file 13148_2024_1658_MOESM2_ESM.docx]

**Table S2: The sequences of primers for qRT-PCR and construction of plasmids**

| Genes | Sequences |
| --- | --- |
| miR-182-5P-RT | 5′-GTC GTA TCC AGT GCA GGG TCC GAG GTA TTC GCA CTG GAT ACG ACA GTG TGA G-3′ |
| miR-182-5P-L | 5′-GAT TTG GCA ATG GTA GAA CTC AC-3′ |
| miR-182-5P-R | 5′-GTG CAG GGT CCG AGG T-3′ |
| U6-RT | 5′-CGC TTC ACG AAT TTG CGT GTC AT-3′ |
| U6-L | 5′-GCT TCG GCA GCA CAT ATA CTA AAA T-3′ |
| U6-R | 5′-CGC TTC ACG AAT TTG CGT GTC AT-3′ |
| PBX3-L | 5′-GAT GCA GCT CAA ACA AAG CAC-3′ |
| PBX3-R | 5′-AGT TAC GCC TTT TCC GTC TGG-3′ |
| BCL2-L | 5′-GGT GGG GTC ATG TGT GTG G-3′ |
| BCL2-R | 5′-CGG TTC AGG TAC TCAG TCA TCC-3′ |
| GAPDH-L | 5′-CCG GGA AAC TGT GGC GTG ATG G-3′ |
| GAPDH-R | 5′-AGG TGG AGG AGT GGG TGT CGC TGT T-3′ |
| Scramble miR-182 sequence | 5′-ACG GAC ATG AGT ACT TCG TAC TAT-3′ |
| *SiCHECK-2- PBX3-L | 5′-CCG CTC GAG CAG GAA GTG TGC ACT CGG AT-3′ |
| *SiCHECK-2- PBX3-R | 5′-ATA AGA ATG CGG CCG CAC AGG GAT AGG CAA ACA GCT-3′ |
| *SiCHECK-2-BCL2-L | 5′-CCG CTC GAG ACC ATG AAA CAA AGC TGC AGG-3′ |
| *SiCHECK-2-BCL2-R | 5′-ATA AGA ATG CGG CCG CAC AGG AGT TTT GAT GGG ACT GT-3′ |
| *MSCV-miR-182-L | 5′-GAA GAT CTT AGG GAT GGT GTC TGC TCC A-3′ |
| *MSCV-miR-182-R | 5′-GGA ATT CAG AGT GTC ACT TCC AGC TGC-3′ |
| ^#^Primer1 of miR-182 genotype | 5′-GGA CCA TAC AGG CCG AAG GAC-3′ |
| ^#^Primer2 of miR-182 genotype | 5′-CCT TCT ATC GCC TTC TTG ACG AGT TC-3′ |
| ^#^Primer3 of miR-182 genotype | 5′-CCC AAG TCC TTT TCA CCG AGA AGA G-3′ |
| ^&^MSP-L | 5′-GTT ATT TAG TTT TAC GGG TGC GT-3′ |
| ^&^MSP-R | 5′-CTT AAA ACA AAT TCC TTT ACG TTC G-3′ |
| ^&^UMSP-L | 5′-GGG TTA TTT AGT TTT ATG GGT GTG T-3′ |
| ^&^UMSP-R | 5′-AAA ACA AAT TCC TTT ACA TTC ACT-3′ |
| ^%^Bis-seq-L | 5′-TTT TTT TAT TTT TAT AGG TAA GGT G-3′ |
| ^%^Bis-seq-R | 5′-TAA AAC CAC TAA AAC AAA TTT CTC C-3′ |

*Primers for construction of plasmids.

^#^Primers for miR-182 KO mice genotype.

^&^Primers for methylation specific PCR (MSP) and unmethylation specific PCR (UMSP) analysis.

^%^Primers for bisulfite-sequencing analysis.
