# Supplementary material for: Low expression of miR-182 caused by DNA hypermethylation accelerates acute lymphocyte leukemia development by targeting PBX3 and BCL2: miR-182 promoter methylation is a predictive marker for hypomethylation agents + BCL2 inhibitor venetoclax
Source: Clin Epigenetics. 2024 Mar 26;16:48. doi: 10.1186/s13148-024-01658-2 (PMC10964616; doi:10.1186/s13148-024-01658-2)
Supplement: Supplementary file 3 — Additional file 3. Supplemental material and methods. [file 13148_2024_1658_MOESM3_ESM.docx]

**Supplemental material and methods**

**Chemical drugs**

BCL-2 inhibitor Venetoclax (Ven, TargetMol, Boston, MA, USA), 5-Azacitidine (AZA, Sigma-Aldrich, St. Louis, MO, USA), 5-aza-2'-deoxycytidine (Decitabine, DAC, Sigma-Aldrich), and puromycin (TargetMol) were dissolved in dimethyl sulphoxide (DMSO, TargetMol) and kept at -20℃ until use.

**Western blot**

Western blot analysis was performed using standard techniques. Briefly, human and murine leukemic cells were harvested and lysed by 1×SDS lysis buffer with phenylmethylsulfonyl fluoride, sodium orthovanadate, and protease inhibitors (Thermo Scientific, Waltham, MA, USA). Then, proteins were extracted and heated at 100℃ for 5 min, followed by rapid cooling at 4℃ for 5 min three times. BCA assay (Thermo Scientific) was performed to measure protein concentration. Equal proteins (20 μg/well) were fractionated by electrophoresis and transferred to PVDF membranes (0.45 μm, Bio-Rad, Richmond, CA, USA). After block by 5% milk for 2 h at room temperature, blots were incubated with primary antibodies overnight at 4 °C, followed by incubation with a secondary HRP-conjugated antibody for 1 h. Blots were washed, and signals were measured by chemiluminescence reagents (Bio-Rad) with an imaging system (Bio-Rad). The following antibodies were used: PBX3 (ab109173, Abcam, Cambridge, MA, USA); BCL2 (ab182858, Abcam). As necessary, blots were stripped and reprobed with β-actin antibody (ab6276, Abcam) or GAPDH (ab8245, Abcam) as endogenous controls.

**Cell proliferation**

Cell proliferation was measured by CCK-8 assay (TargetMol). Briefly, ALL cells (1×10^4^/per well) were plated on 96-well plates and were incubated with 10 μL CCK-8 solution for 4 h in an incubator with 5% CO_2_. OD values were measured at 450 nm by a spectrophotometer (PerkinElmer Health Sciences, Waltham, MA, USA).

**Cell viability assay**

Cell viability was measured by trypan-blue (Gibco) staining and automatic cell counter (Countess 3, Thermo Scientific).

**Apoptosis assays**

Apoptosis was measured by Annexin V/7-AAD staining (Invitrogen). Briefly, ALL cells were collected and washed by 1×binding buffer. Cells were incubated with 100 μL 1×binding buffer containing 5.0 μL Annexin V-APC and 5.0 μL 7-AAD at room temperature for 20 min. Leukemic cells were resuspended with 400 μL 1×binding buffer and analyzed by flow cytometry within 30 min (CytoFLEX LX, Beckman Coulter).

**Luciferase activity**

HeLa cells were plated on 24-well plate one day before transfection. Each well was transiently co-transfected with psiCHECK-2 plasmid carrying the wide-type 3'-untranslated region (UTR) of *BCL2* and *PBX3* or 3'UTR with the mutated miR-182-binding sites of *BCL2* and *PBX3*, together with 50 pmol miR-182 mimics (GenePharma, Shanghai, China) or scramble (SCR). All cell lysates were harvested, and Firefly and Renilla luciferase activities were measured by the Dual-Luciferase Reporter Assay two days after transfection (Promega, Madison, WI, USA).

**Retroviral and lentiviral production and transduction**

For the production of retrovirus and lentivirus, HEK293T cells (4×10^6^) were plated in a 10 cm dish one day before transfection. Retroviral vector MSCV together with packaging plasmids (Gap pol and VSVG) and lentiviral vector LVX together with packaging plasmids (MD2G and PSPA2) were co-transfected into 293T cells together with 45 μg linear polyethylenimine (Sigma-Aldrich). The virus was collected from the supernatant at 48 and 72 h after transfection and filtered by a 0.45 μm polysulfone filter (Millipore, Billerica, MA, USA).

**Colony formation**

Murine GFP^+^ ALL cells were sorted and plated on murine methylcellulose medium (MethoCult™ GF M3434, Stemcell Technologies, Vancouver, BC, Canada), and colonies were counted ten days after plating according to manufacturer's protocol.

Transfection of scrambled miR-182 (SCR182)

Before transfection, ALL cells were seeded in 6-well plates at a density of 2.0×10^5^/ml per well. 100 pmol SCR182 (GenePharma, Shanghai, China) or blank control (Ctrl) was transiently transfected into ALL cell lines by Lipofectamine 3000 transfection reagent (Invitrogen). Cells were collected for proliferation and apoptosis analysis at the indicated times after transfection.

**H&E staining**

Paraformaldehyde-fixed paraffin-embedded sections of murine spleen and liver tissues were subjected to H&E staining by standard protocols.

**RNA sequencing (RNA-seq)**

BM GFP^+^ cells from 182WT and 182KO ALL mice were sorted by flow cytometry. Total RNA was extracted by Trizol reagent (Invitrogen, USA) according to the manufacturer's protocol. mRNA enrichment, fragmentation, and cDNA synthesis were performed by KAPA Stranded RNA-Seq Library Preparation Kit (Illumina Technologies, San Diego, CA, USA) to complete library construction. Sequencing was performed on Illumina HiSeq 4000 sequencing platform. Illumina/Solexa Pipeline Image analysis was performed to analyze base calling and error estimation. To reconstruct the transcriptome, the trimmed reads were mapped to the corresponding reference genome by HISAT2 and StringTie. DESeq algorithm was used to filter the differentially expressed genes. *P* value and FDR analysis are under the following criteria (Fold Change >2 or <0.5 and FDR < 0.05). Pathway analysis was used to find out the significant pathway of the differential genes according to KEGG database. The raw data of the RNA-seq experiments has been deposited in GEO (GSE260974).

**Construction of plasmids**

We amplified human pre-miR-182 and its flanking regions and cloned it into retroviral vector pMSCV-puro (Clontech, Palo Alto, CA, USA) to produce the plasmid expressing human miR-182. miR-182-binding base sites at the 3'-UTR of *PBX3* and *BCL2* were amplified by PCR and subsequently cloned and inserted into the psiCHECK-2 vector (Promega). The mutations in the miR-182-binding sites in the 3'-UTR of *PBX3* and *BCL2* were generated by a site-directed mutagenesis kit (Stratagene, Santa Clara, CA, USA). All of the primer sequences are shown in Table S2. DNA sequencing was performed to confirm the sequence.

**Flow cytometry analysis**

BM cells isolated by crushing bones from mice were collected and stained with various primary antibodies in the staining buffer for 30 min. Antibodies include B220-PE (BD Pharmingen, San Diego, CA, USA), CD43-APC (BD Pharmingen), CD3-PC5.5 (BD Pharmingen), Gr-1-APC (BD Pharmingen), and Mac-1-PC7 (BD Pharmingen). Thymidine analog 5-ethynyl-2'-deoxyuridine (EdU) staining was used to determine the proliferation status of ALL cells following the manufacturer's protocol (Beyotime Biotechnology). Analysis was performed by Flow cytometry (CytoFLEX LX, Beckman-Coulter), and cell sorting was performed by a FACSAria II (Becton Dickinson). The Data were analyzed with FlowJo software v10.0 (Ashland, OR, USA).

**Luciferase activity**

On the day before transfection, Hela cells (1×10^5^/mL per well) were plated on 24-well plates. Each well was transiently co-transfected with 100 ng psiCHECK-2 plasmid bearing the wide-type 3'-untranslated region (UTR) of *PBX3* and *BCL2* or 3'UTR with the mutated miR-182-binding site of *PBX3* and *BCL2* for 24 h, together with 60 pmol scramble (SCR), or miR-182 mimics (GenePharma, Shanghai, China) for another 24 h. Cell lysates were collected after the transfection for 48 h. Firefly and Renilla luciferase activities were measured by the Dual-Luciferase Reporter Assay System (Promega, Madison, WI, USA). Firefly luciferase activity was normalized to that of Renilla luciferase as relative luciferase activity for each assay.

**Engraftment of NOD/SCID‑IL2Rγ mice (NSG)**

Busulfan (30 mg/kg; Sigma) was intraperitoneally injected to 8-week-old male NSG mice (Shanghai Model Organisms Center, Shanghai, China) one day before transplantation. Total of 1×10^6^ 182OE-NALM-6 or 182NC-NALM-6 cells were xenografted by vein injection into NSG mice. hCD45 frequency was measured in blood when 182NC-NALM-6 cells-xenografted mice demonstrated signs of illness. Survival time was determined from the first day of the experiment until death.
