# Supplementary figures and images for: Low expression of miR-182 caused by DNA hypermethylation accelerates acute lymphocyte leukemia development by targeting PBX3 and BCL2: miR-182 promoter methylation is a predictive marker for hypomethylation agents + BCL2 inhibitor venetoclax
Source: Clin Epigenetics. 2024 Mar 26;16:48. doi: 10.1186/s13148-024-01658-2 (PMC10964616; doi:10.1186/s13148-024-01658-2)

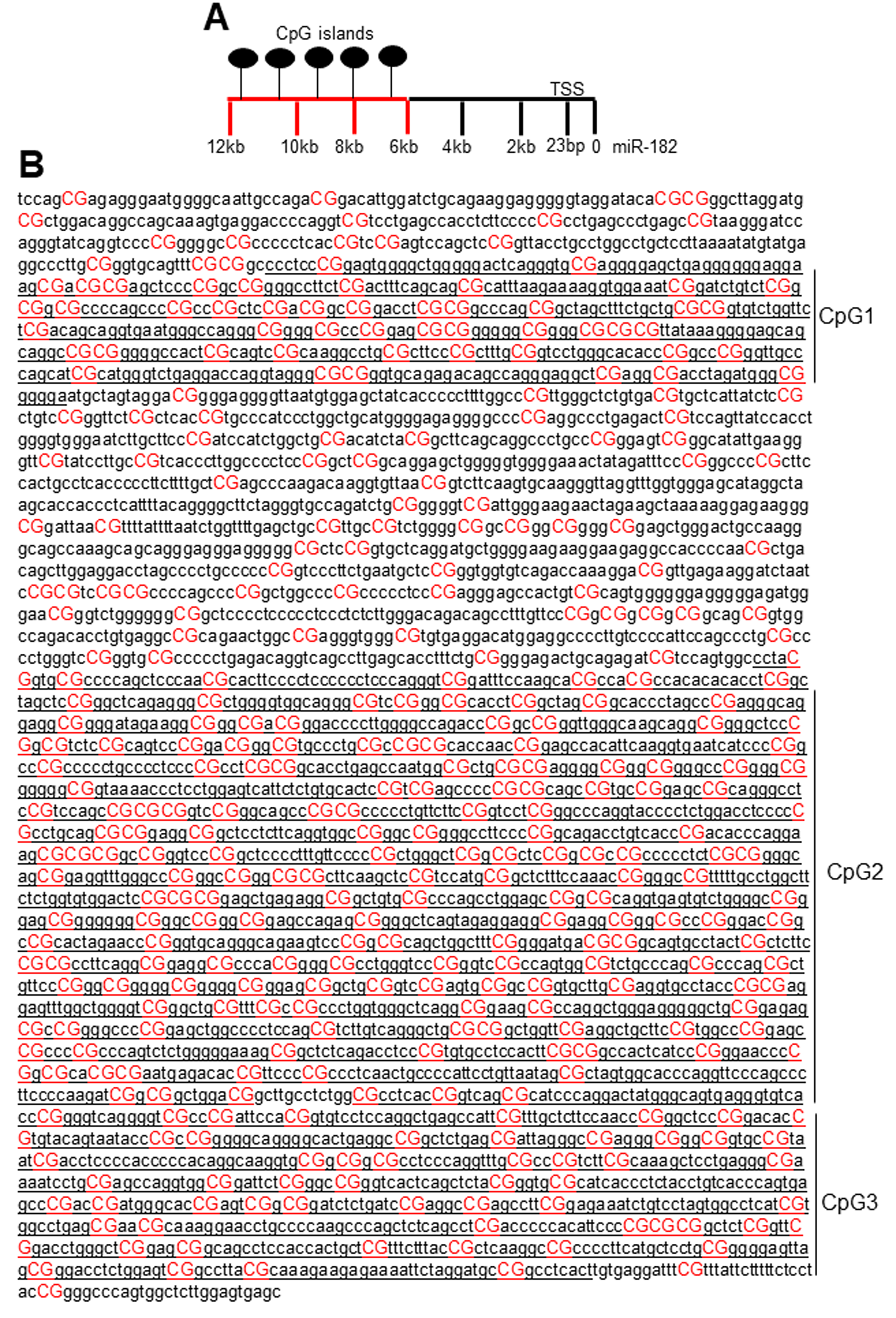

Supplement: Supplementary file 4 — Additional file 4: Fig. S1. The detailed three CpG islands at miR-182 promoter. (A) The regions indicating the CpG islands at the miR-182 promoter. TSS: transcription start site. (B) The detailed base information of three CpG islands [file 13148_2024_1658_MOESM4_ESM.tif]

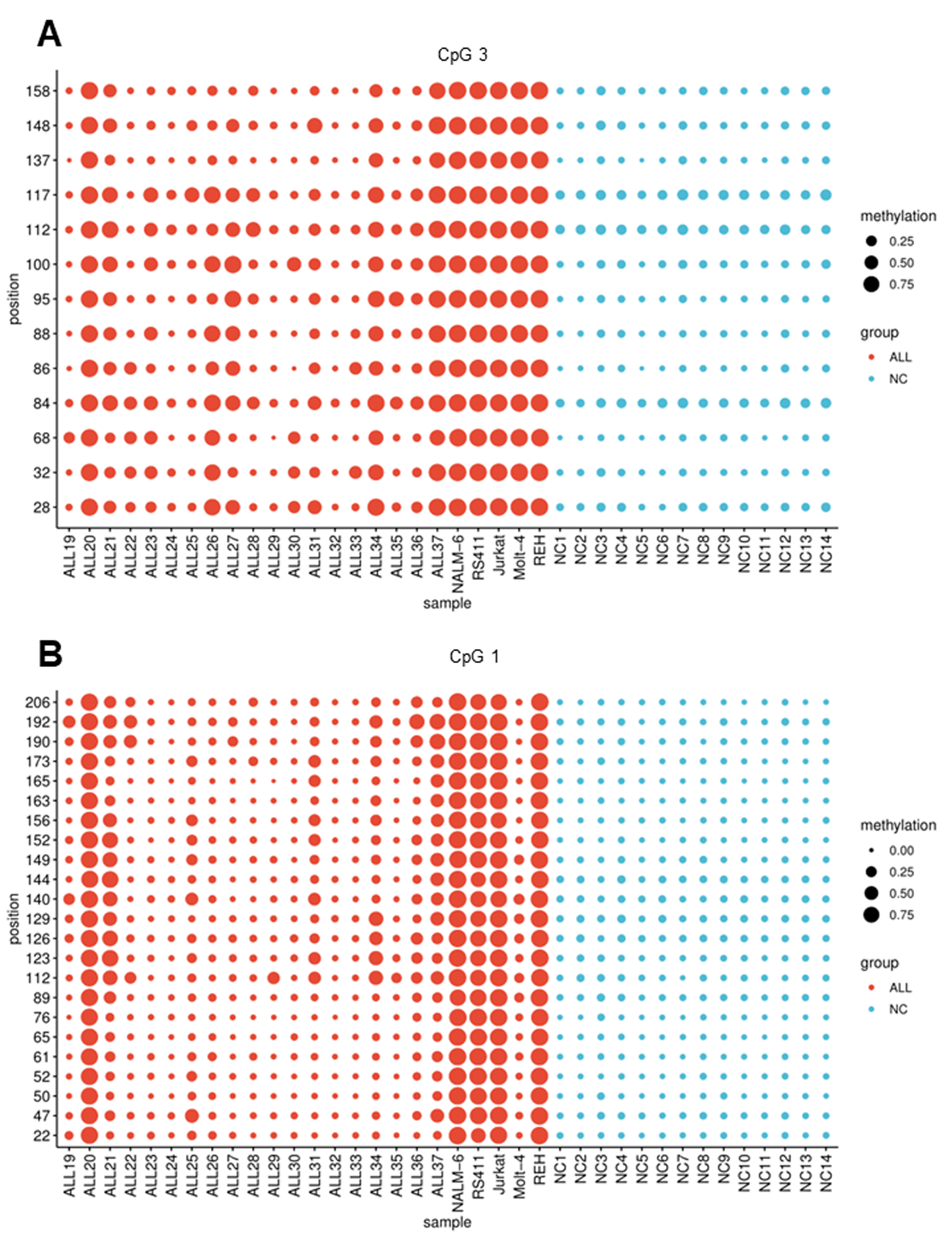

Supplement: Supplementary file 5 — Additional file 5: Fig. S2. The methylation frequency of CpG island 3 and 1 at the miR-182 promoter by MethylTargetTM assay. (A and B) The detail methylation of CpG island 3 and 1 in 14 NCs, 19 primary ALL blasts, and 5 ALL cell lines [file 13148_2024_1658_MOESM5_ESM.tif]

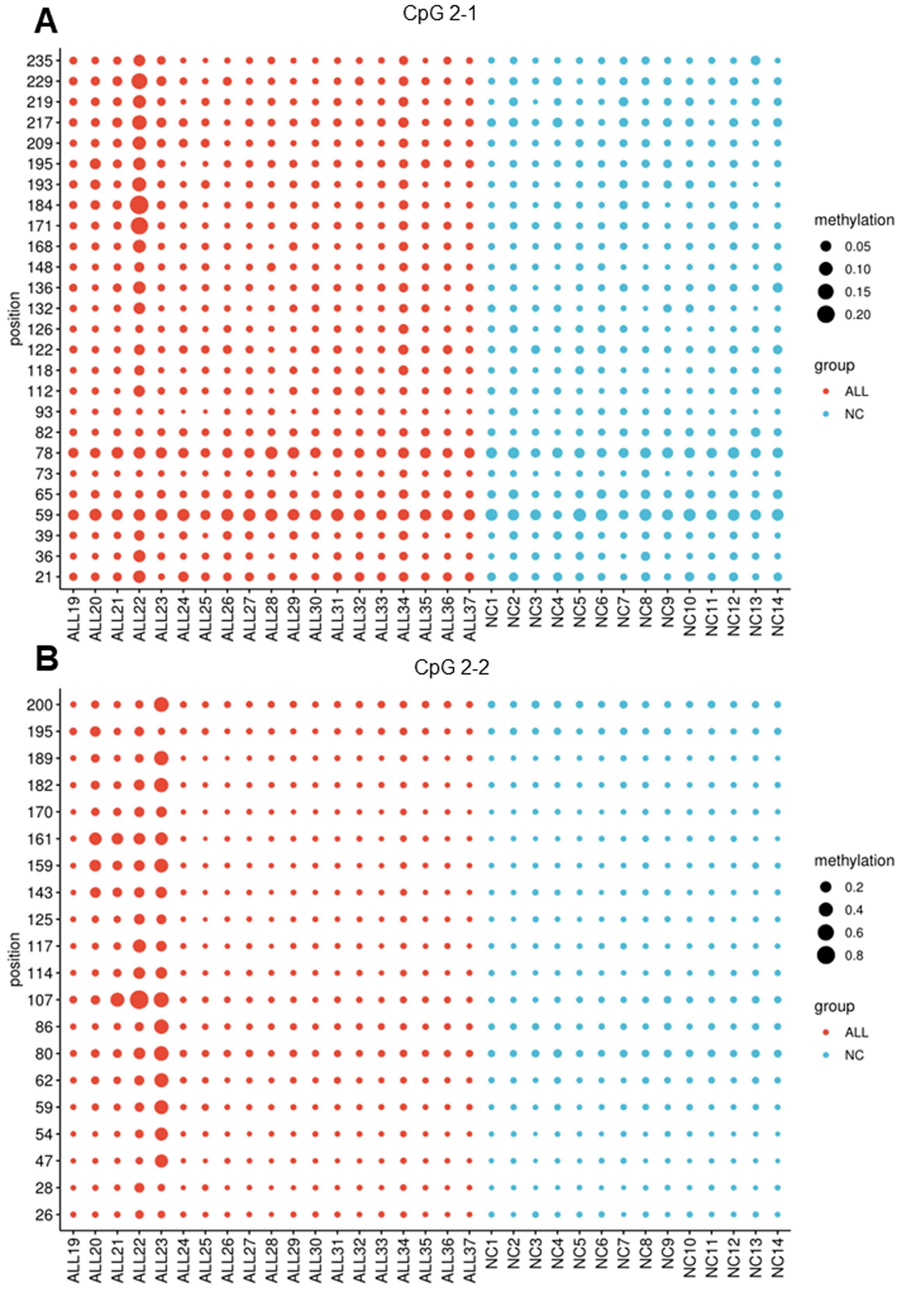

Supplement: Supplementary file 6 — Additional file 6: Fig. S3. The methylation frequency of CpG island 2 at the miR-182 promoter by MethylTargetTM assay. (A and B) The detailed methylation information of CpG island 2 in 14 NCs and 19 primary ALL blasts [file 13148_2024_1658_MOESM6_ESM.tif]

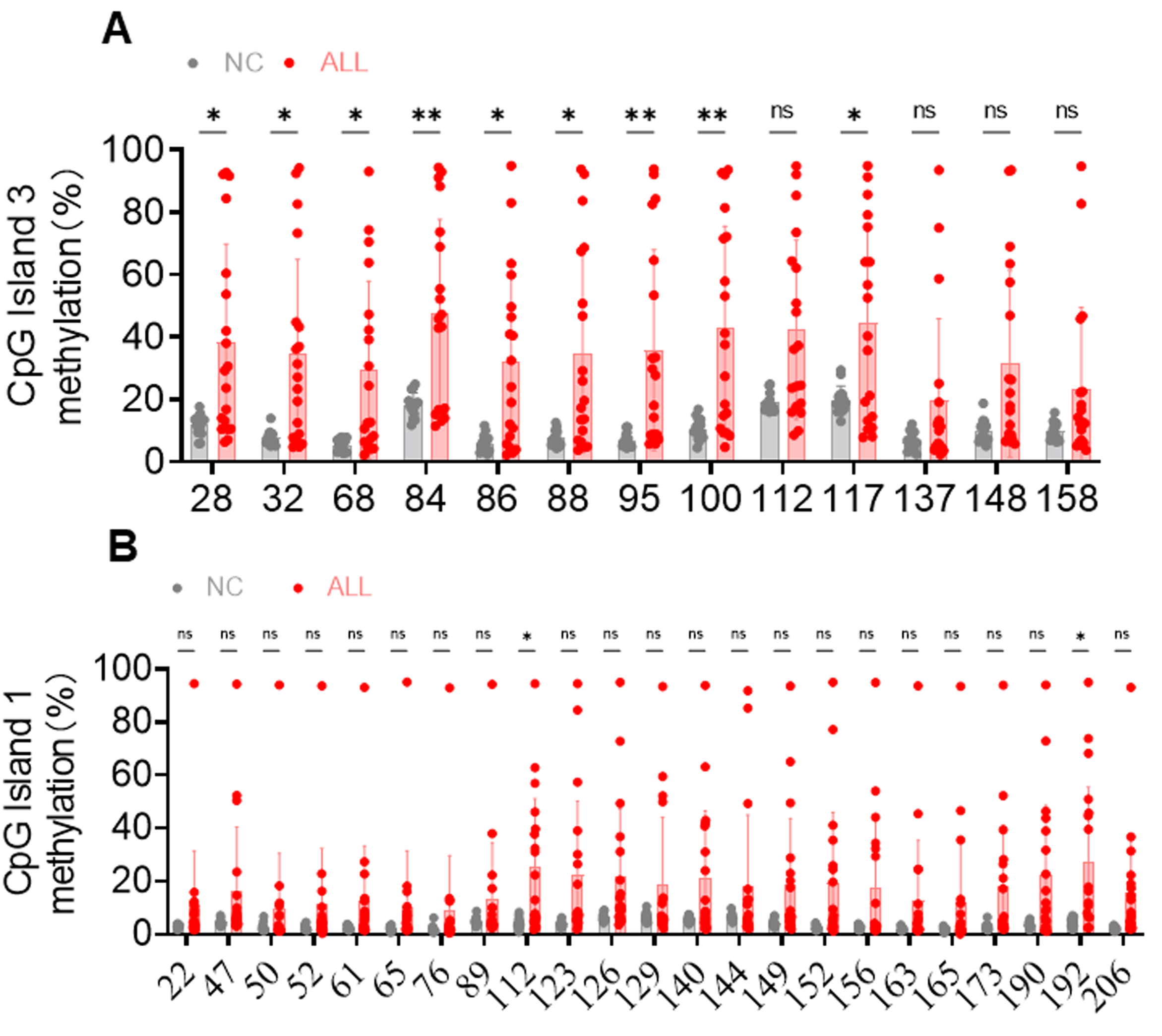

Supplement: Supplementary file 7 — Additional file 7: Fig. S4 Individual site analysis of methylation frequency in CpG island 3 and 1. (A and B) Individual site analysis of methylation frequency in CpG island 3 (A) and 1 (B) was performed in 14 NCs and 19 primary ALL blasts [file 13148_2024_1658_MOESM7_ESM.tif]

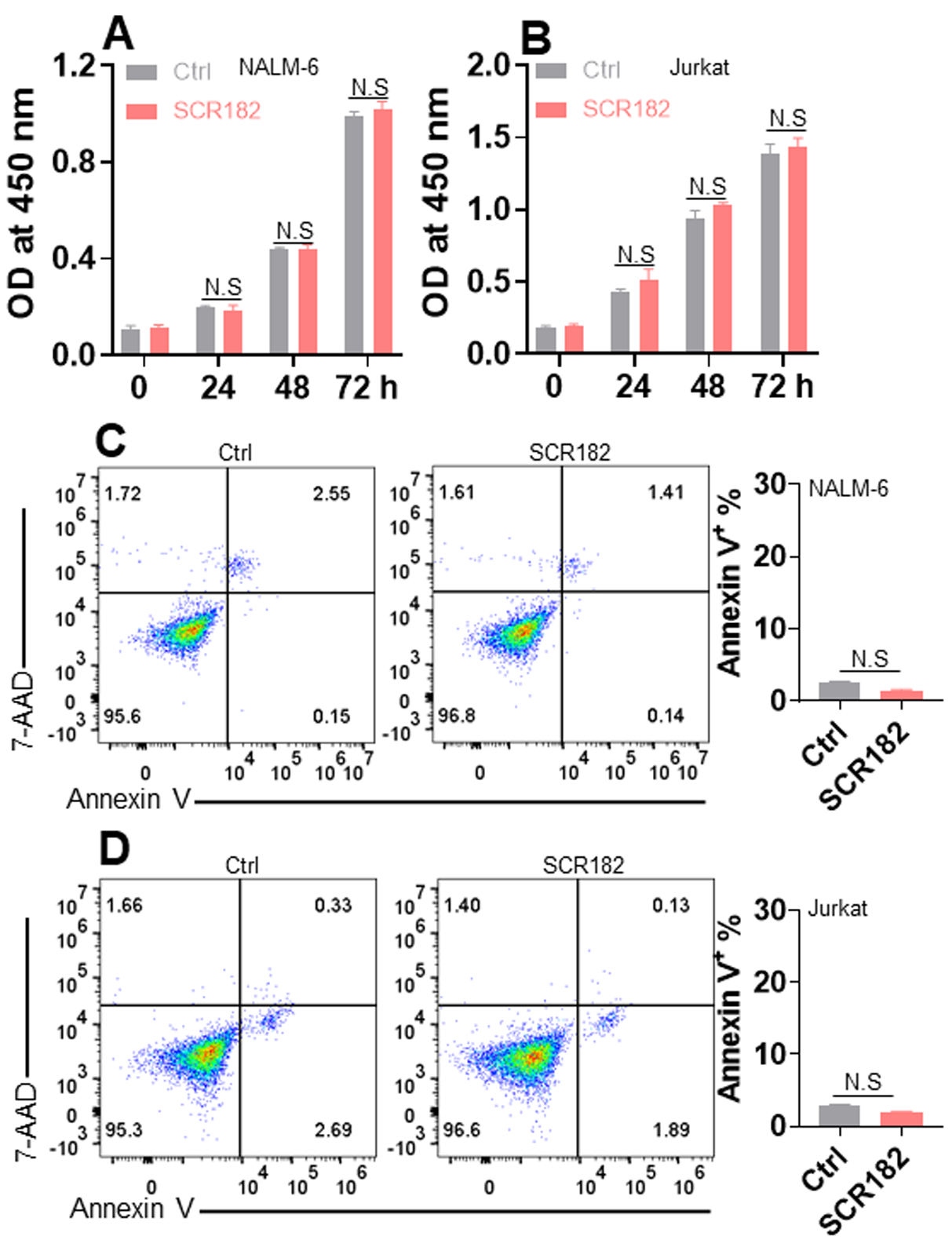

Supplement: Supplementary file 8 — Additional file 8: Fig S5. Overexpression of a scrambled miR-182 (SCR182) does not affect cell proliferation and apoptosis in ALL cells. (A and B) CCK8 activity was measured in NALM-6 and Jurkat cells transfected with SCR182 or blank control (Ctrl) for the indicated days. (C and D) Apoptosis was measured in NALM-6 and Jurkat cells transfected with SCR182 or Ctrl for 48 h by Annexin V and 7-AAD staining. Representative flow cytometry plots (left) and statistical analysis of the percentage of Annexin V+ cells (right) are shown. N.S: not significant [file 13148_2024_1658_MOESM8_ESM.tif]

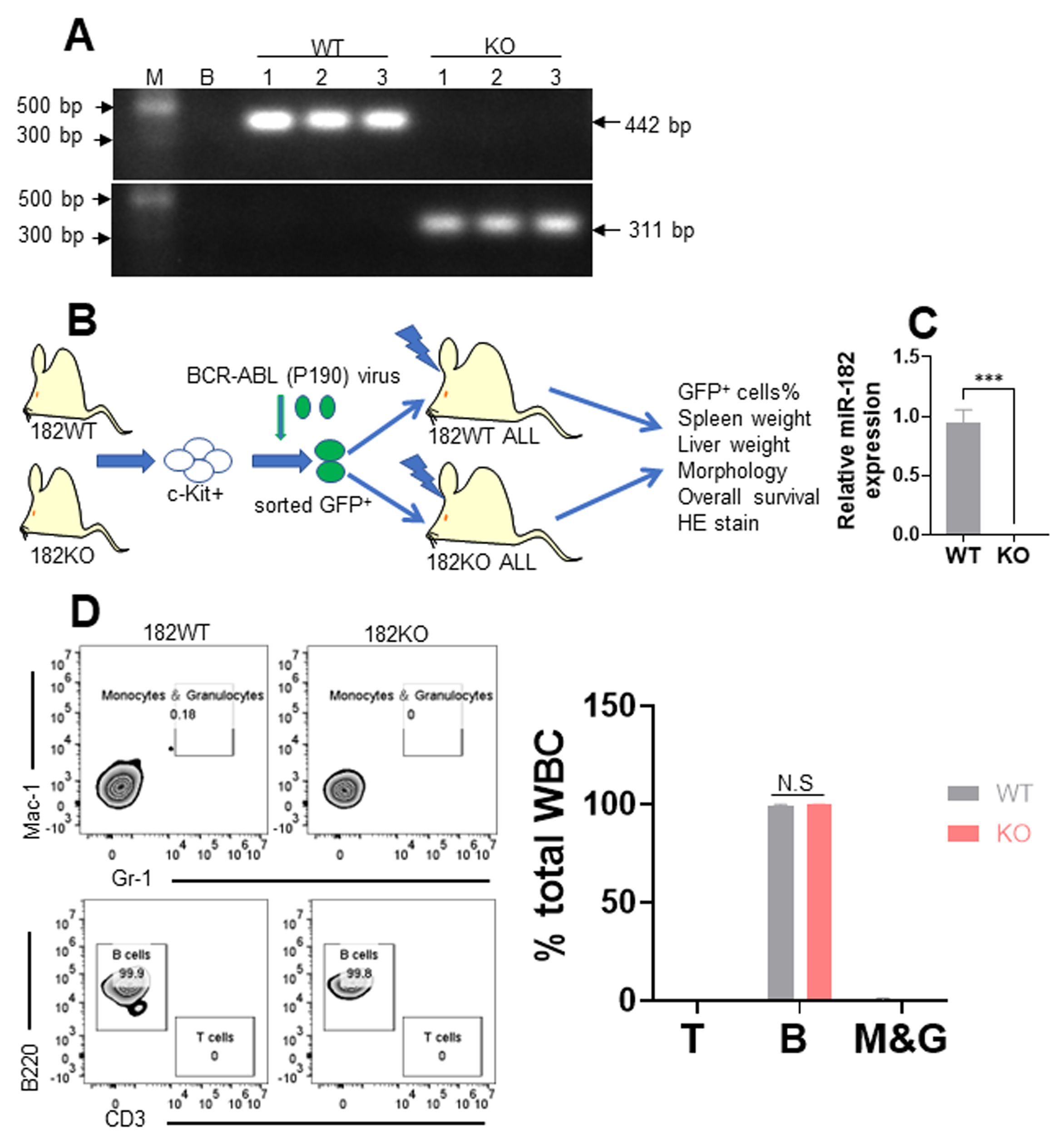

Supplement: Supplementary file 9 — Additional file 9: Fig S6. Genotype of WT mice (182WT) and miR-182-knockout (182KO) mice and schedule for BCR-ABL (P190)-induced B-ALL mouse model. (A) Genotyping PCR for 182WT and 182KO mice. (B) Schedule for BCR-ABL (P190)-induced B-ALL mouse model. (C) Murine miR-182 expression was measured by qRT-PCR in BM GFP+ cells from 182WT B-ALL mice and 182KO B-ALL mice. (D) CD3, B220, Mac-1, and Gr-1 expressions were measured in BM GFP+ cells from primary 182WT and 182KO B-ALL mice by flow cytometry. Shown are the representative flow cytometry plots (left) and statistical analysis of T lymphocyte (CD3+), B lymphocyte (B220+), monocyte and granulocyte (Mac-1+ or Gr-1+) (Right). ***P<0.001; N.S: not significant [file 13148_2024_1658_MOESM9_ESM.tif]

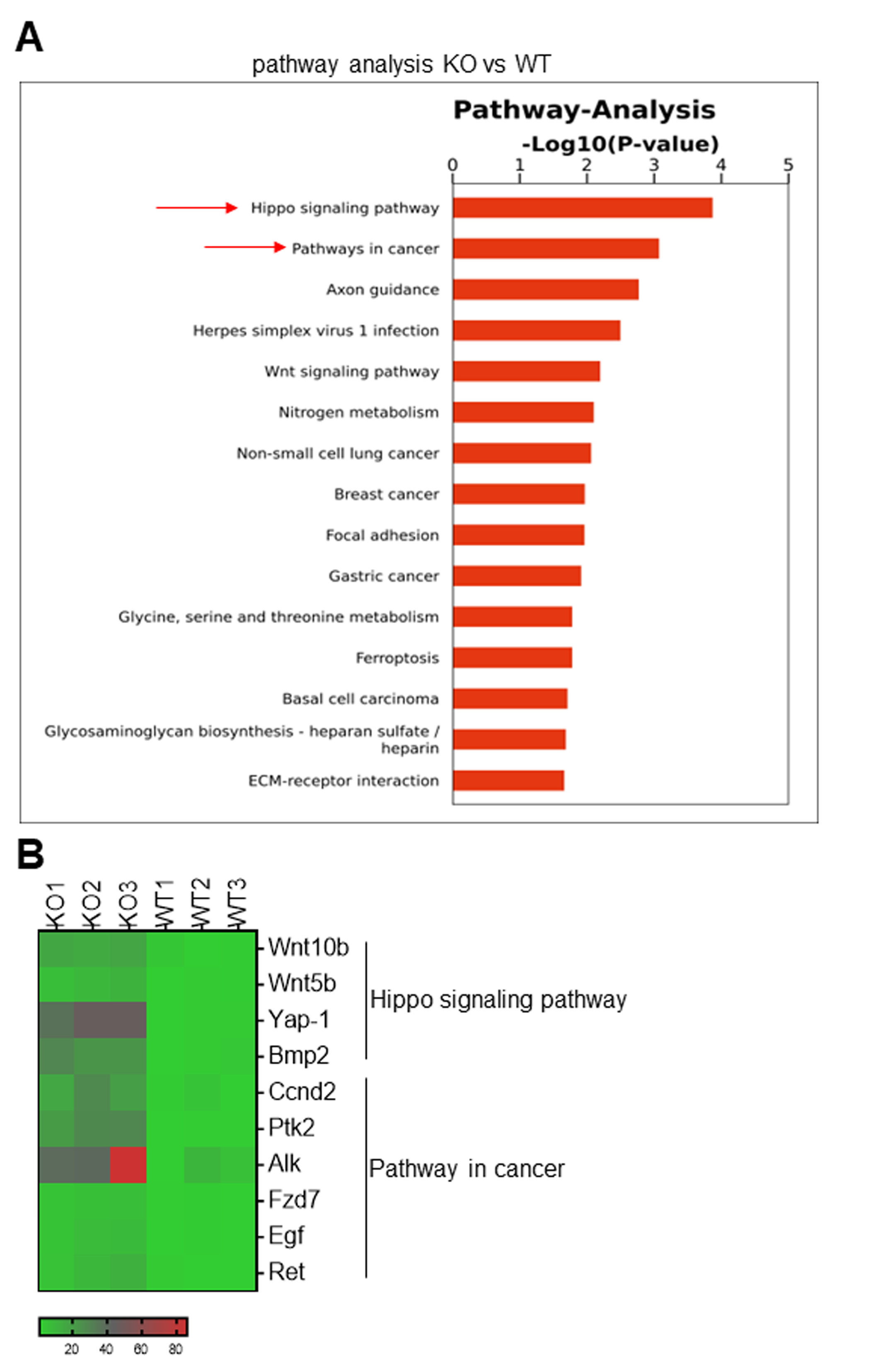

Supplement: Supplementary file 11 — Additional file 11: Fig S7. RNA-seq for BM GFP+ cells from 182WT B-ALL mice and 182KO B-ALL mice. (A and B) Pathway analysis demonstrated that Hippo signaling, including Wnt and Yap-1, and pathway in cancer, including Ccnd2 and Fzd7, were enriched in 182KO B-ALL cells compared with 182WT B-ALL cells [file 13148_2024_1658_MOESM11_ESM.tif]

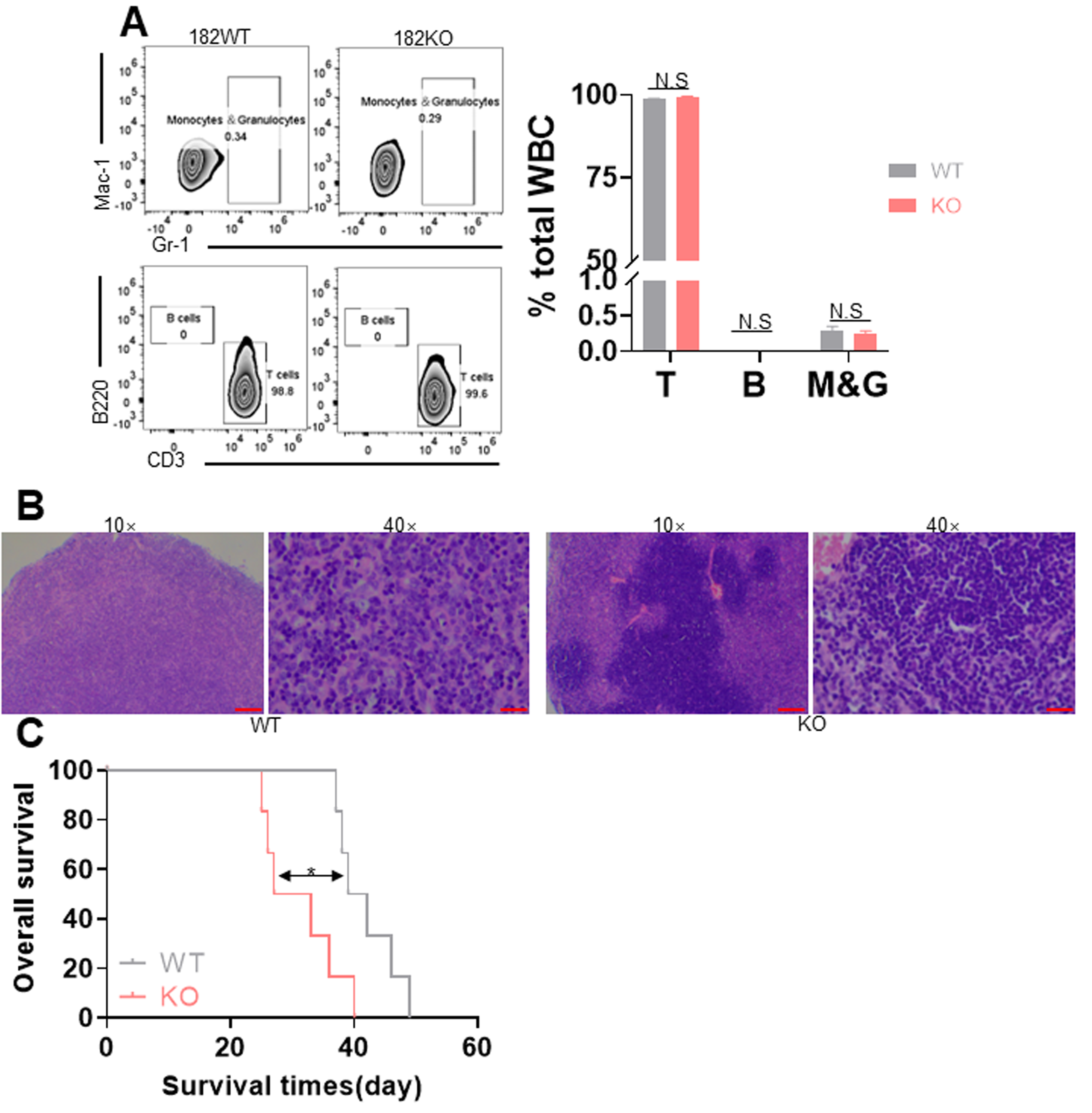

Supplement: Supplementary file 12 — Additional file 12: Fig S8. Depletion of miR-182 accelerates the development of murine Notch-transformed T-ALL model. (A) CD3, B220, Mac-1, and Gr-1 expressions were measured in BM GFP+ cells from 182WT and 182KO T-ALL mice. Shown are the representative flow cytometry plots (left) and statistical analysis of T lymphocyte (CD3+), B lymphocyte (B220+), monocyte, and granulocyte (Mac-1+ and Gr-1+). (B) HE staining of thymus tissue from 182WT and 182KO T-ALL mice. Bar for 10× is 200 μm and for 40× is 20 μm. (C) OS was analyzed in primary BMT (N= 6 for WT and KO). *P<0.05; N.S: not significant [file 13148_2024_1658_MOESM12_ESM.tif]

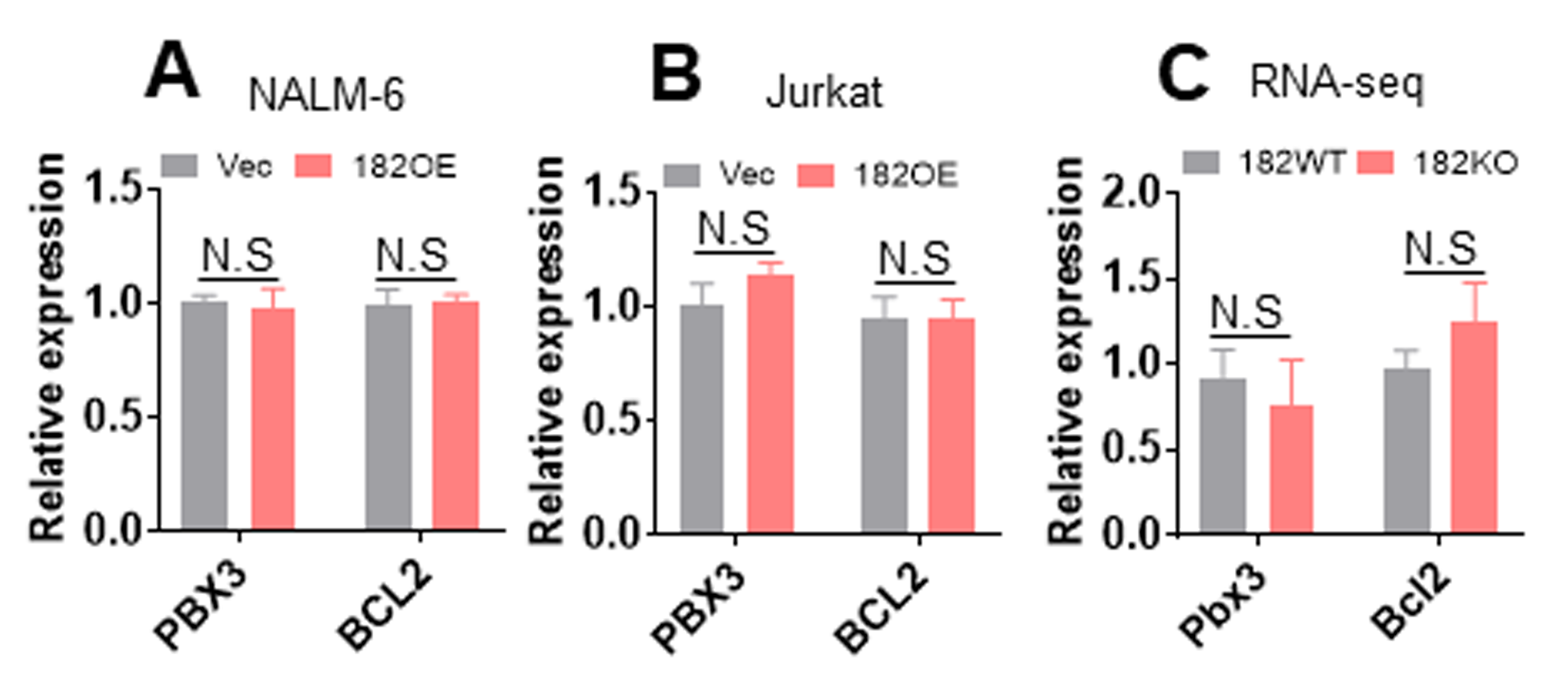

Supplement: Supplementary file 13 — Additional file 13: Fig S9. Overexpression of miR-182 does not significantly affect PBX3 and BCL2 transcript levels. (A and B) The transcript levels of PBX3 and BCL2 were measured in NALM-6 and Jurkat cells transduced with MSCV-miR-182 overexpressing miR-182 (182OE) or blank vector (Vec). (C) The relative Pbx3 and Bcl2 transcript levels were analyzed in BM GFP+ cells from murine 182WT and 182KO B-ALL model according to RNA-seq analysis. N.S: not significant [file 13148_2024_1658_MOESM13_ESM.tif]

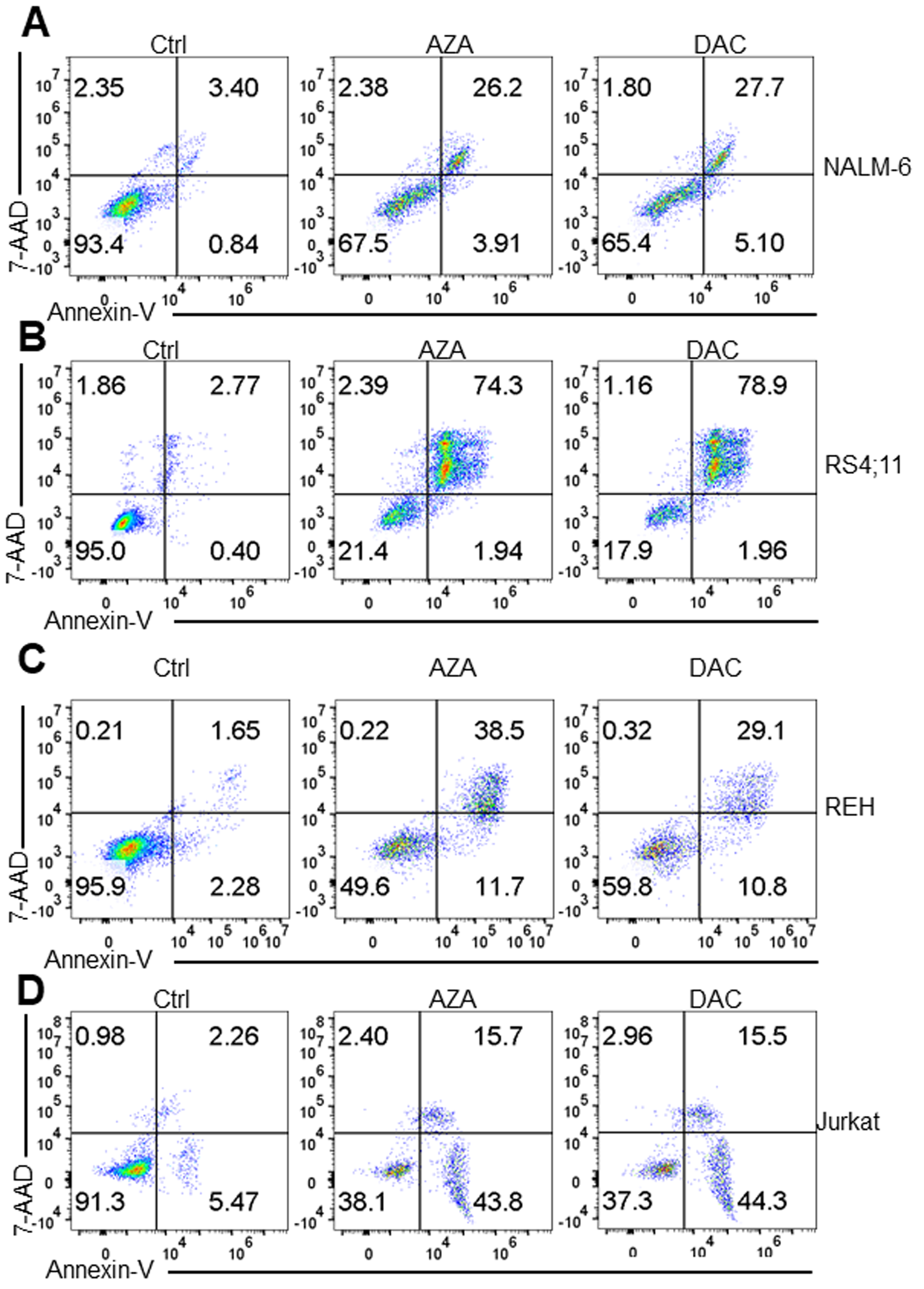

Supplement: Supplementary file 14 — Additional file 14: Fig S10. HMAs induce apoptosis in ALL cells. (A-D) Apoptosis was measured by Annexin V/7-AAD staining in four ALL cell lines treated with or without DAC (5 μM) or AZA (5 μM) for four days. Representative flow cytometry plots were shown [file 13148_2024_1658_MOESM14_ESM.tif]
