# Supplementary material for: Low expression of miR-182 caused by DNA hypermethylation accelerates acute lymphocyte leukemia development by targeting PBX3 and BCL2: miR-182 promoter methylation is a predictive marker for hypomethylation agents + BCL2 inhibitor venetoclax
Source: Clin Epigenetics. 2024 Mar 26;16:48. doi: 10.1186/s13148-024-01658-2 (PMC10964616; doi:10.1186/s13148-024-01658-2)
Supplement: Supplementary file 10 — Additional file 10. Limiting dilution assay of BCR-ABL (P190)-transduced 182WT and 182KO B-ALL mice. [file 13148_2024_1658_MOESM10_ESM.docx]

**Table S3. Limiting dilution assay of BCR-ABL (P190)-transduced 182WT and 182KO B-ALL mice.**

| Dose | 182WT (response/total) | 182KO (response/total) | *P* value |
| --- | --- | --- | --- |
| 10 | 2/7 | 5/7 |  |
| 100 | 3/7 | 7/7 |  |
| 1000 | 7/7 | 7/7 |  |
| LSC frequency | 1 in 117.99 | 1 in 8.49 | <0.001 |

The numbers of response mice mean that the recipient mice develop full-blown leukemia and die within six months after transplantation.
